# Clks 1, 2 and 4 prevent chromatin breakage by regulating the Aurora B-dependent abscission checkpoint

**DOI:** 10.1038/ncomms11451

**Published:** 2016-04-29

**Authors:** Eleni Petsalaki, George Zachos

**Affiliations:** 1 Department of Biology, University of Crete, Vassilika Vouton, Heraklion, Crete 70013, Greece

## Abstract

When chromatin is trapped at the intercellular bridge, cells delay completion of cytokinesis (abscission) to prevent chromosome breakage. Here we show that inhibition of Cdc-like kinases (Clks) 1, 2 or 4 accelerates midbody resolution in normally segregating cells and correlates with premature abscission, chromatin breakage and generation of DNA damage in cytokinesis with trapped chromatin. Clk1, Clk2 and Clk4 localize to the midbody in an interdependent manner, associate with Aurora B kinase and are required for Aurora B–serine 331 (S331) phosphorylation and complete Aurora B activation in late cytokinesis. Phosphorylated Aurora B–S331 localizes to the midbody centre and is required for phosphorylation and optimal localization of the abscission protein Chmp4c. In addition, expression of phosphomimetic mutants Aurora B–S331E or Chmp4c-S210D delays midbody disassembly and prevents chromatin breakage in Clk-deficient cells. We propose that Clks 1, 2 and 4 impose the abscission checkpoint by phosphorylating Aurora B–S331 at the midbody.

## Abstract (web summary)

Cells delay completion of cytokinesis when chromatin is trapped at the intercellular bridge. Here, Petsalaki and Zachos report that Cdc-like kinases (Clks) 1, 2 and 4 localize to the midbody and phosphorylate the mitotic kinase Aurora B, imposing the abscission checkpoint to prevent premature abscission and chromatin breakage.

## Graphical abstract

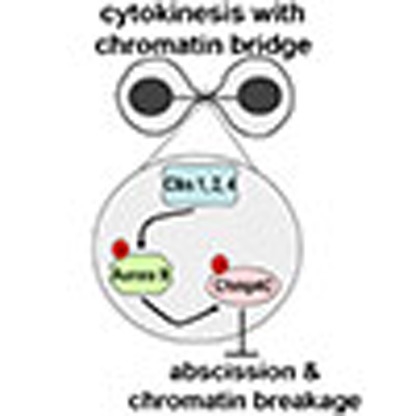

Chromatin bridges represent incompletely segregated chromosomal DNA connecting the anaphase poles or daughter nuclei and have been linked to chromosomal instability in human tumours and tumourigenesis in mice^1^,^2^. In response to chromatin bridges or to lagging chromosomes that are trapped in the intercellular bridge in late cytokinesis, eukaryotic cells delay abscission, the final cut of the narrow cytoplasmic canal that connects the daughter cells, to prevent chromosome breakage or tetraploidization by regression of the cleavage furrow^3^,^4^,^5^,^6^. In mammals, this abscission delay is called ‘the abscission checkpoint' and is dependent on Aurora B kinase^5^. Aurora B localizes to the midbody and imposes the abscission checkpoint by phosphorylating the endosomal sorting complex required for transport-III (ESCRT-III) subunit charged multivesicular body protein 4C (Chmp4c) on serines 210, 214 and 215 in human cells^6^,^7^. This phosphorylation has been proposed to target Chmp4c to the midbody centre, to prevent downstream endosomal sorting complex required for transport components including the ATPase Vps4 from relocalizing to the abscission site and deliver the final cut^6^,^7^,^8^,^9^. In addition, in normally segregating cells, that is, in the absence of trapped chromatin at the intercellular bridge, inhibition of Aurora B accelerates abscission, suggesting that the abscission checkpoint may function more generally as an abscission timer^5^,^6^. However, the mechanism of Aurora B activation in the abscission checkpoint is a matter of active investigation.

Complete Aurora B kinase activity requires phosphorylation at S331 (ref. 10). The DNA damage kinases Chk1 and Chk2 phosphorylate Aurora B–S331 in mitosis: Chk2 phosphorylates Aurora B–S331 in early prometaphase, while Chk1 phosphorylates S331 in late prometaphase and metaphase^11^,^12^,^13^. However, the kinase that activates Aurora B in the late stages of cytokinesis has not been previously reported.

The Cdc-like kinases (Clk1–4 in human cells) are an evolutionary conserved family of dual specificity protein kinases, which can autophosphorylate at tyrosine residues and phosphorylate their substrates on serine/threonine residues^14^,^15^. Clks localize in the cytoplasm and in the nucleus where they regulate alternative splicing through phosphorylation of serine/arginine-rich domains on splicing factors^16^,^17^,^18^. Clks recognize the minimum consensus sequence R-x-x-S/T also shared by Chk1 and Chk2; however, our current understanding of Clk biological targets and function is relatively limited^15^,^19^,^20^.

In the present study, we show that depletion of Clk1, Clk2 or Clk4 by small interfering RNA (siRNA) or pharmacological inhibition of Clk catalytic activity accelerates midbody resolution in normally segregating human cells. Furthermore, Clk-deficient cells exhibit premature abscission, chromatin breakage and generation of DNA damage in cytokinesis with chromatin bridges. Clks 1, 2 and 4 phosphorylate Aurora B–S331 *in vitro* and are required for optimal Aurora B–phosphorylation and complete Aurora B activation in late cytokinesis. In addition, Clk1, Clk2 and Clk4 localize to the midbody in an interdependent manner and associate with Aurora B in cell extracts after enrichment of cells in cytokinesis. Using cells transiently expressing siRNA-resistant forms of wild-type (WT) or phosphomimetic S331E Aurora B after depletion of the endogenous protein, we propose that Clk-dependent Aurora B–S331 phosphorylation is required for phosphorylation and optimal localization of Chmp4c to the midbody centre in late cytokinesis, in the absence or the presence of DNA bridges. In addition, expression of S331E Aurora B or overexpression of the phosphomimetic mutant S210D Chmp4c delays midbody disassembly and prevents chromatin breakage in Clk-deficient cells. On the basis of these findings, we propose that Clk1, Clk2 and Clk4 impose the abscission checkpoint by phosphorylating Aurora B–S331 at the midbody.

## Results

### Clk inhibition accelerates midbody disassembly

**Figure 1:**
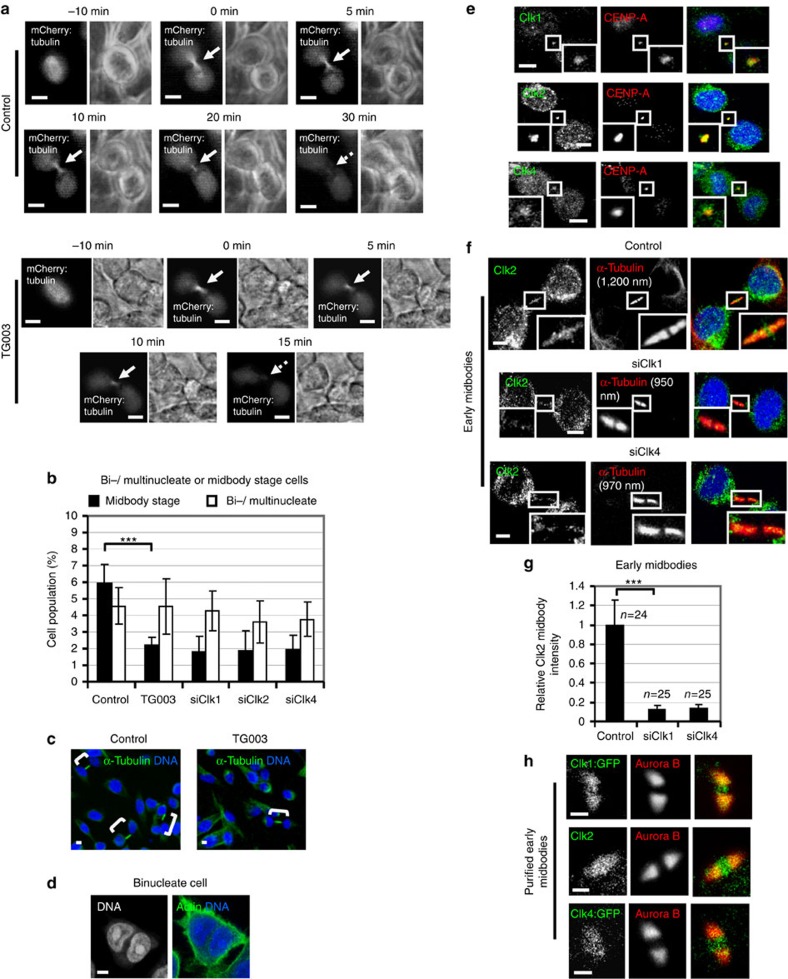
Clk inhibition accelerates midbody disassembly in normally segregating cells. (**a**) Midbody disassembly in the absence (control) or in the presence of TG003. Cells expressing mCherry:tubulin were analysed by time-lapse microscopy. Phase-contrast (right panels) and fluorescence images (left panels) are shown. Time from midbody formation to midbody disassembly is indicated. Midbodies are shown by solid arrows and midbody disassembly is indicated by broken arrows. (**b**) Frequency of bi-/multinucleate or midbody stage cells. Cells were transfected with negative siRNA (control), Clk1 siRNA (siClk1), Clk2 siRNA (siClk2), Clk4 siRNA (siClk4) or treated with TG003 for 5 h. Error bars show the s.d. from the mean from three independent experiments. A minimum of 300 cells were analysed per experiment. ***P<0.001 compared with control. The Student's *t*-test was used. (**c**) Examples of midbody stage cells (shown in brackets). Green, α-tubulin; blue, DNA. (**d**) Example of a binucleate cell after TG003 treatment. Green, actin; blue, DNA. (**e**) Localization of Clk1, Clk2 or Clk4 in cytokinesis. Green, Clk1, Clk2 or Clk4; red, CENP-A; blue, DNA. Twenty cells from two independent experiments were examined. (**f**) Localization of Clk2 in cells transfected as in **b**. Green, Clk2; red, α-tubulin; blue, DNA. Tubulin values indicate midbody thickness. Insets show magnified midbodies. (**g**) Mean midbody intensity of Clk2 in cells treated as in **f**. Data are from *n* cells from three independent experiments. Error bars show s.d. Values in control were set to 1. ***P<0.001 compared with control. The Mann–Whitney *U*-test was used. (**h**) Localization of Clk1:GFP, Clk2 or Clk4:GFP in purified midbodies. Green, GFP or Clk2; red, Aurora B. Scale bars, 5 μm.

To investigate a role for Cdc-like kinases in midbody resolution, human colon carcinoma BE cells transiently expressing α-tubulin fused to mCherry (mCherry:tubulin) were monitored by time-lapse microscopy and the kinetics of tubulin disassembly at the midbody determined (Fig. 1a)^21^. In control cells, the midbody remained visible for a median time of 35±5 min after formation (*n*=9; Supplementary Movies 1 and 2). In contrast, treatment of cells with 1 μM TG003, an inhibitor of Clk1, Clk2 and Clk4 catalytic activity at this concentration^22^, accelerated midbody disassembly (*t*=18±3 min, *n*=8) compared with controls (*P*<0.001; Supplementary Movies 3 and 4). This correlated with reduced frequency of cells at midbody stage after treatment with TG003 or depletion of Clk1, Clk2 or Clk4 by two independent siRNAs, but not with an increase in binucleate or multinucleate cells compared with controls (Fig. 1b–d and Supplementary Figs 1a–f and 2b). Furthermore, Clk-deficient and control cells exhibited similar frequency of cells in prometaphase (Supplementary Fig. 2a,b), suggesting that Clk inhibition does not prevent mitotic entry and that Clk-deficient cells can progress through abscission and disassemble their midbodies more rapidly than controls. We propose that Clks 1, 2 and 4 regulate proper timing of midbody resolution in normally segregating cells.

### Clks localize to the midbody

We then investigated localization of Clk1, Clk2 and Clk4 by immunofluorescence using CENP-A as a midbody marker (Supplementary Fig. 2c–e)^23^,^24^. We found that Clk1, Clk2 and Clk4 localized to the midbody in normally segregating cells (Fig. 1e).

During cytokinesis, the microtubule bundles at the midbody progressively get narrower^6^,^25^ and microscopic examination showed that midbody thickness ranges from ∼400 to 1,400 nm in BE cells (*n*=100). To further investigate localization of Clks in cytokinesis, ‘early' or ‘late' midbodies exhibiting midbody thickness of 800–1,400 or 400–700 nm, respectively, were examined. In control cells, Clk1 fused to green fluorescent protein (GFP) (Clk1:GFP) and Clk2 or Clk4 fused to GFP (Clk4:GFP) proteins localized on midbody arms and inside the Flemming body, that is, the narrow region in the midbody centre where tubulin staining by immunofluorescence is blocked^26^, in early or late midbodies, and they partially co-localized with Aurora B (Fig. 1f,h and Supplementary Fig. 2f). Depletion of Clk1 or Clk4 diminished localization of Clk2 to early or late midbodies compared with controls (Fig. 1f,g and Supplementary Fig. 2f). These results show that Clks 1, 2 and 4 localize to the midbody in normally segregating cells, and that Clk1 and Clk4 are required for the localization of Clk2 in the structure.

### Clks phosphorylate Aurora B-S331 at the midbody

**Figure 2:**
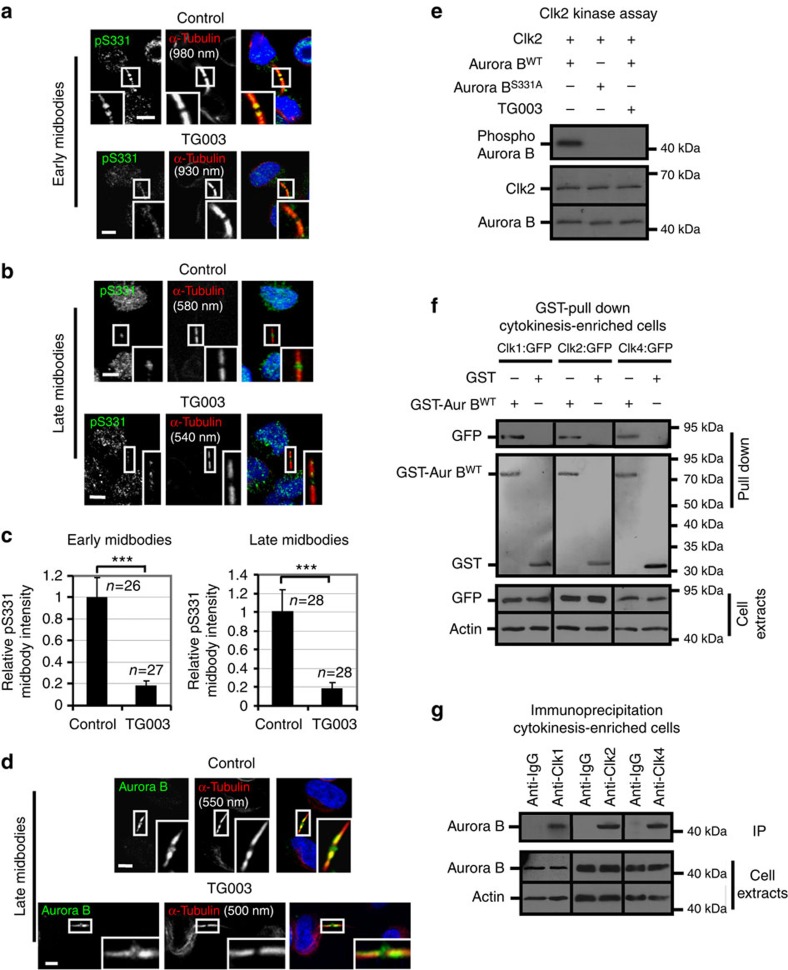
Aurora B associates with Clks in cell lysates. (**a**,**b**) Aurora B–S331 phosphorylation (pS331) in the absence (control) or presence of TG003 for 5 h. Green, pS331; red, α-tubulin; blue, DNA. (**c**) Mean midbody intensity of pS331 in cells treated as in **a**,**b**. Data are from *n* cells from three independent experiments. Values in control were set to 1. Error bars show s.d. ***P<0.001 compared with control. The Mann–Whitney *U*-test was used. (**d**) Localization of total Aurora B in cells treated as in **b**. Green, Aurora B; red, α-tubulin; blue, DNA. Thirty cells from three independent experiments were examined per treatment. Tubulin values indicate midbody thickness. Insets show magnified midbodies. Scale bars, 5 μm. (**e**) Clk2 *in vitro* kinase assay. Autoradiography analysis of Aurora B substrates (top) and Ponceau staining (bottom). (**f**) GST pull-down assay from cytokinesis-enriched cells. Lysates expressing Clk1:GFP, Clk2:GFP or Clk4:GFP proteins were incubated with 10 μg glutathione–agarose-bound WT GST–tagged Aurora B (GST-Aur B^WT^) or GST. Associated GFP (top) or total GFP and actin (bottom) were detected by western blotting and GST proteins (middle) by Ponceau staining. (**g**) Immunoprecipitation assay from cytokinesis-enriched cells. Immunoprecipitated (IP) Aurora B (top) and total Aurora B and actin (bottom) were detected by western blotting.

Recruitment of active Aurora B at the midbody delays abscission^5^. We therefore investigated Aurora B–S331 phosphorylation, a marker of Aurora B activation, at the midbody^10^. In early midbodies, phosphorylated Aurora B–S331 localized on midbody arms in control cells where it partially overlapped with midbody microtubules (Fig. 2a). Inhibition of Clk catalytic activity by TG003 diminished Aurora B–S331 phosphorylation (Fig. 2a,c); however, it did not prevent localization of total Aurora B on midbody arms compared with control cells (Supplementary Fig. 2g). Surprisingly, in late midbodies, phosphorylated Aurora B–S331 localized inside the Flemming body in control cells (Fig. 2b). Treatment of cells with TG003 or depletion of Clk1, Clk2, Clk4 or Aurora B by siRNA reduced phospho-Aurora B–S331 staining in the Flemming body compared with controls (Fig. 2b,c and Supplementary Fig. 3a,b). The majority of total Aurora B localized on midbody arms in late midbodies; however, a relatively small proportion of total Aurora B was also detectable in the midbody centre in Clk-deficient or control cells (Fig. 2d and Supplementary Fig. 3c). These results show that Clks 1, 2 and 4 are required for Aurora B–S331 phosphorylation at the midbody in normally segregating cells. In contrast, inhibition of Chk1 using the Chk1-selective inhibitor UCN-01 or depletion of Chk2 by siRNA did not reduce Aurora B–S331 phosphorylation at the midbody compared with control cells (Supplementary Fig. 3d,e).

Recombinant proteins Clk1, Clk2 or Clk4 phosphorylated Aurora B in an *in vitro* kinase assay and substrate phosphorylation was remarkably reduced after mutation of S331 to alanine (S331A) or inhibition of Clks by TG003 compared with WT or kinase-dead (KD) Aurora B (Fig. 2e and Supplementary Fig. 4a–c). Transiently expressed Clk1:GFP, Clk2:GFP or Clk4:GFP proteins associated with glutathione *S*-transferase (GST)–Aurora B, but not with GST, in pull-down assays after enrichment of cells in cytokinesis (Fig. 2f and Supplementary Fig. 4d). Furthermore, Aurora B was precipitated from cell extracts using antibodies against endogenous Clk1, Clk2 or Clk4 after enrichment of cells in cytokinesis but not from asynchronous cells (Fig. 2g and Supplementary Fig. 4e), suggesting that Clks 1, 2 and 4 interact with Aurora B in cytokinesis, but not in interphase. In addition, Clk4 was precipitated from cell extracts from cytokinesis-enriched cells using antibodies against Clk1 or Clk2, and Clk2 was precipitated by antibodies against Clk1 or Clk4, which is consistent with Clks 1, 2 and 4 participating in the same protein complexes (Supplementary Fig. 4f,g). Taken together, we propose that Clk1, Clk2 and Clk4 associate with Aurora B and phosphorylate Aurora B–S331 at the midbody in normally segregating cells.

### Clks regulate localization of Chmp4c to late midbodies

**Figure 3:**
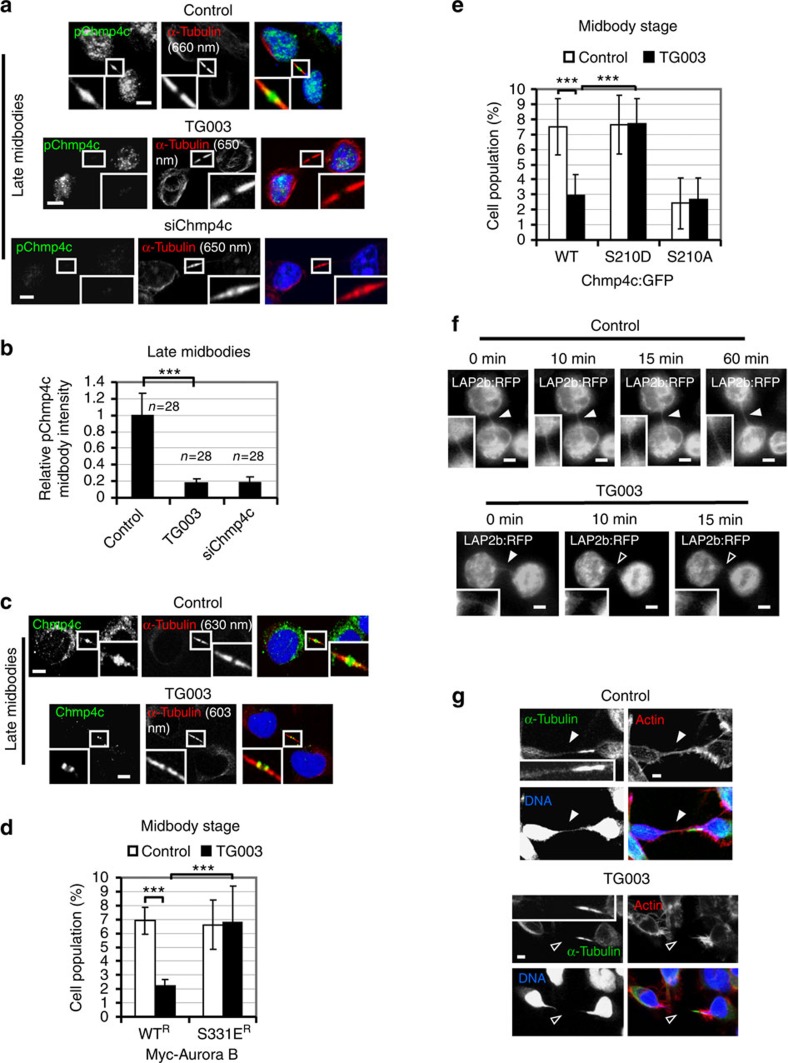
Expression of the phosphomimetic S331E Aurora B or S210D Chmp4c mutants rescues the frequency of cells at midbody stage after Clk inhibition. (**a**) Localization of phosphorylated Chmp4c-S210, S214 and S215 (pChmp4c) in the absence (control) or presence of TG003 for 5 h, or after transfection of cells with Chmp4c siRNA (siChmp4c). Green, pChmp4c; red, α-tubulin; blue, DNA. (**b**) Mean midbody intensity of pChmp4c in cells treated as in **a**. Data are from *n* cells from three independent experiments. Error bars show s.d. Values in control were set to 1. ***P<0.001. The Mann–Whitney *U*-test was used. (**c**) Localization of total Chmp4c in cells treated as in **a**. Green, Chmp4c; red, α-tubulin; blue, DNA. Thirty cells from three independent experiments were examined per treatment. Tubulin values indicate midbody thickness. Insets show magnified midbodies. (**d**,**e**) Frequency of cells at midbody stage. (**d**) Cells expressing siRNA-resistant forms of WT or S331E Myc-tagged Aurora B (Myc-Aurora B) were depleted of endogenous Aurora B by siRNA and treated as in **a**. (**e**) Cells expressing WT, S210D or S210A Chmp4c:GFP were treated as in **a**. Error bars show the s.d. from the mean from three independent experiments. A minimum of 200 cells were analysed per experiment. ***P<0.001. Student's *t*-test was used. (**f**) Breakage of intercellular bridges after Clk inhibition. HeLa cells expressing LAP2b:RFP were analysed by time-lapse microscopy in the absence (control) or presence of TG003. Time from formation of the LAP2b:RFP bridge is indicated. Higher magnification insets of LAP2b:RFP bridges are shown. (**g**) Intercellular bridges in fixed BE cells treated as in **a**. Thirty-five cells from three independent experiments were examined per treatment. Green, α-tubulin; red, actin; blue, DNA. Higher magnification insets of tubulin staining are shown. Intact intercellular or chromatin bridges are indicated by solid arrowheads and broken bridges by open arrowheads. Scale bars, 5 μm.

Aurora B phosphorylates Chmp4c to delay abscission^6^,^7^. To investigate Chmp4c phosphorylation at the midbody, an antibody against phosphorylated S210, S214 and S215 of human Chmp4c (pChmp4c) was used^7^. In control cells, phosphorylated Chmp4c localized on midbody arms in early midbodies and inside the Flemming body in late midbodies (Fig. 3a and Supplementary Fig. 5a). Inhibition of Clk catalytic activity by TG003 or depletion of Chmp4c by siRNA (siChmp4c) diminished Chmp4c phosphorylation in late midbodies compared with control cells (Fig. 3a,b). However, Chmp4c or Aurora B protein levels *per se* were not affected by TG003-treatment (Supplementary Fig. 5b). In addition, phosphorylation of Chmp4c in early midbodies after Clk inhibition was similar to control cells (Supplementary Fig. 5a,b). As Clk inhibition reduced Aurora B–S331 phosphorylation in early midbodies as described in the previous section, one possibility is that there is redundancy in phosphorylation of Chmp4c at this stage, and that an unidentified kinase can phosphorylate Chmp4c at midbody arms when Aurora B activity is diminished. In late midbodies, total Chmp4c localized inside the Flemming body in control cells but mislocalized on midbody arms in TG003-treated cells (Fig. 3c). Importantly, this antibody recognized Chmp4c but not Chmp4a or Chmp4b proteins (Supplementary Fig. 5c). These results show that Cdc-like kinase activity is required for optimal phosphorylation and localization of Chmp4c to late midbodies in normally segregating cells.

### Aurora B–S331 phosphorylation delays midbody resolution

To investigate the significance of Aurora B–S331 phosphorylation, BE cells transiently expressing Myc-tagged, siRNA-resistant versions of WT (WT^R^) or a phosphomimetic S331 to glutamic acid (S331E^R^) mutant Aurora B were analysed after depletion of the endogenous Aurora B by siRNA (Supplementary Fig. 5d). Expression of S331E^R^, but not WT^R^, Myc-Aurora B restored the frequency of cells at midbody stage after Clk inhibition by TG003 compared with controls, whereas mitotic entry, judged by the frequency of cells in prometaphase, was similar in all treatments (Fig. 3d and Supplementary Fig. 5e). These results suggest that phosphorylated Aurora B–S331 delays midbody disassembly in Clk-deficient cells.

To investigate the importance of Chmp4c phosphorylation, WT Chmp4c fused to GFP (WT Chmp4c:GFP), S210D Chmp4c:GFP harbouring a phosphomimetic mutation of S210 to aspartic acid or S210A Chmp4c:GFP containing a non-phosphorylatable mutation of S210 to alanine were transiently expressed at approximate levels tenfold higher than the endogenous protein, to disrupt endogenous Chmp4c functions (Supplementary Fig. 5f). Correct localization of Chmp4c:GFP proteins at the midbody was maintained as will be shown in Supplementary Fig. 9c. Expression of S210D, but not WT, Chmp4c:GFP rescued the frequency of cells at midbody stage after treatment with TG003 compared with controls (Fig. 3e and Supplementary Fig. 5g), suggesting that Chmp4c-S210 phosphorylation prevents faster midbody resolution in Clk-deficient cells compared with control cells. In contrast, expression of S210A Chmp4c:GFP accelerated midbody disassembly in the absence or presence of TG003 as judged by the reduced frequency of cells at midbody stage compared with controls expressing WT Chmp4c:GFP (Fig. 3e and Supplementary Fig. 5g). We propose that proper timing of midbody resolution in normally segregating cells requires Clk-mediated Aurora B–S331 phosphorylation and subsequent Chmp4c-S210 phosphorylation by Aurora B at the midbody.

### Clks prevent chromatin breakage in cytokinesis

To investigate the role for Clks in the abscission checkpoint in the presence of chromatin bridges, HeLa cells stably expressing the inner nuclear envelope marker LAP2b fused to red fluorescent protein (RFP) (LAP2b:RFP) were monitored in cytokinesis by time-lapse microscopy. Gradual thinning of chromosome bridges during mitotic exit limits their detection by time-lapse imaging of chromatin markers; however, LAP2b:RFP localizes around chromatin from late anaphase and always correlates with chromatin bridges in high-resolution still images in which DNA-binding stains are used^5^. Control cells exhibiting LAP2b:RFP intercellular bridges sustained those bridges for at least 60 min (*n*=9; Fig. 3f and Supplementary Movie 5). In contrast, in 11/12 cells treated with the Clk inhibitor TG003, LAP2b:RFP bridges were rapidly ruptured (average time to LAP2b bridge breakage 11±7 min, *n*=11; Fig. 3f, Supplementary Fig. 6a and Supplementary Movies 6 and 7).

**Figure 4:**
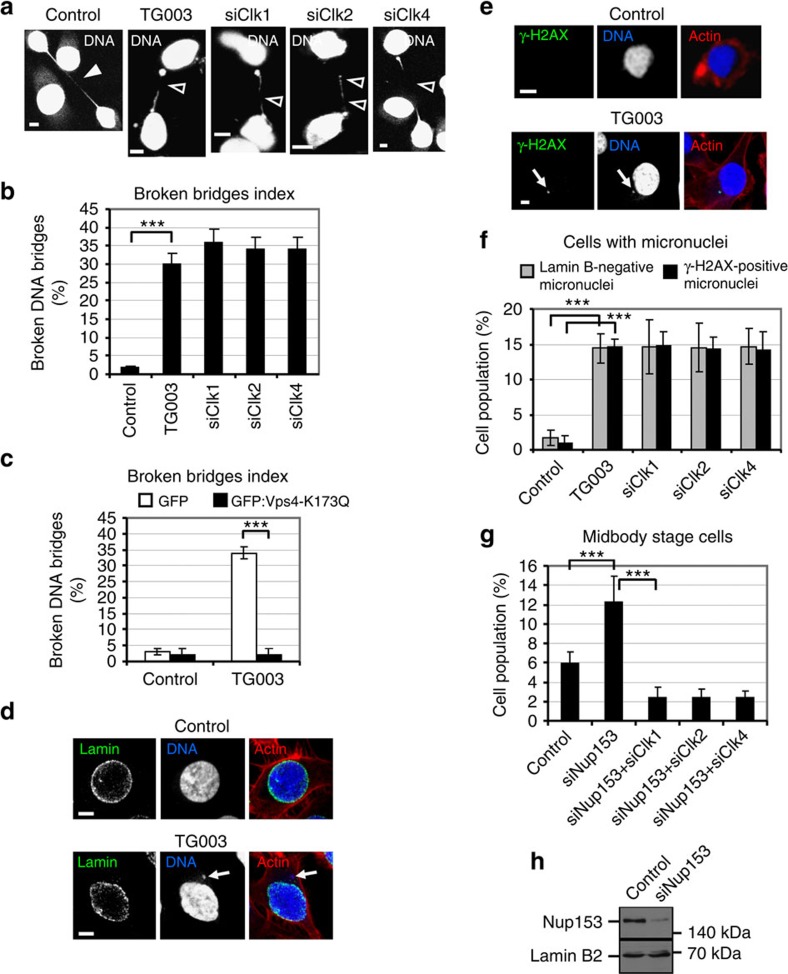
Clk inhibition correlates with chromatin breakage and generation of DNA damage. (**a**) Cells were transfected with negative siRNA (control), Clk1 siRNA (siClk1), Clk2 siRNA (siClk2), Clk4 siRNA (siClk4) or treated with TG003 for 5 h. Intact DNA bridges are indicated by solid and broken bridges by open arrowheads. (**b**) Broken bridges index analysis in cells treated as in **a**. Error bars show the s.d. from the mean from three independent experiments. A minimum of 50 cells with chromatin bridges were analysed per experiment. (**c**) Cells expressing GFP or GFP:Vps4-K173Q were treated as in **a**. Broken bridges index shows the percentage of green cells with broken chromatin bridges/total green cells with chromatin bridges. Error bars show the s.d. from the mean from three independent experiments. A minimum of 50 green cells with chromatin bridges were analysed per experiment. (**d**) Lamin B-negative micronuclei (marked by arrow) in cells treated as in **a**. Green, lamin B2; red, actin; blue, DNA. (**e**) γ-H2AX-positive micronuclei (indicated by arrow) in cells treated as in **a**. Green, γ-H2AX; red, actin; blue, DNA. Scale bars, 5 μm. (**f**) Frequency of cells exhibiting lamin B-negative or γ-H2AX-positive micronuclei after treatment as in **a**. Error bars show the s.d. from the mean from three independent experiments. A minimum of 150 cells were analysed per experiment. (**g**) Frequency of cells at midbody stage. Cells were transfected with negative siRNA (control), Nup153 siRNA (siNup153) or combinations of Nup153 siRNA and Clk1 siRNA (siNup153+siClk1), Nup153 siRNA and Clk2 siRNA (siNup153+siClk2) or Nup153 siRNA and Clk4 siRNA (siNup153+siClk4). Error bars show the s.d. from the mean from three independent experiments. A minimum of 300 cells were analysed per experiment. ***P<0.001 compared with control. Student's *t*-test was used. (**h**) Westen blot analysis of Nup153 and lamin B2 in cells treated as in **g**.

Confocal microscopy analysis of fixed HeLa or BE cells in telophase showed that 30–36% cells treated with TG003 or depleted of Clk1, Clk2 or Clk4 by two independent siRNAs exhibited broken DNA bridges compared with 2–3% controls (Fig. 4a,b and Supplementary Fig. 6b–e). In addition, all broken DNA bridges exhibited broken LAP2b intercellular bridges and vice versa (Supplementary Fig. 6b,c). Furthermore, 35/35 BE control cells with stable chromatin bridges exhibited intact intercellular bridges as evidenced by staining with α-tubulin and actin, and 35/35 TG003-treated cells with broken DNA bridges exhibited broken intercellular bridges, indicating that these cells had completed abscission (Fig. 3g). These results suggest that Clk activity is required to prevent abscission and chromatin breakage in cytokinesis with chromatin bridges.

To further investigate whether broken chromatin bridges were caused by abscission, cells expressing mutant Vps4 protein fused to GFP in which Vps4-lysine 173 was changed to glutamine (GFP:Vps4-K173Q), to abrogate ATP binding to Vps4 and inhibit abscission^9^, or GFP alone were examined. Expression of GFP:Vps4-K173Q prevented chromatin breakage after treatment with TG003 compared with cells expressing GFP (Fig. 4c and Supplementary Fig. 6f), suggesting that chromatin breakage in Clk-deficient cells was caused by abscission.

Fragmented chromatin bridges can lead to the formation of micronuclei and accumulation of DNA damage^1^. Treatment with TG003 or depletion of Clk1, Clk2 or Clk4 increased the frequency of cells exhibiting micronuclei compared with controls and these micronuclei were devoid of nuclear lamin B2, which is consistent with being bridge derived (Fig. 4d,f)^27^. This increase in lamin B-negative micronuclei in Clk-deficient cells was not due to an overall increase in the frequency of cells with chromatin bridges (both intact and broken) compared with controls (Supplementary Fig. 7a). Furthermore, in Clk-deficient cells, micronuclei were positive for phospho-Ser139 histone H2A.X (γ-H2AX) staining, which is a marker for double-strand DNA breaks (Fig. 4e,f)^28^. These results show that Clks 1, 2 and 4 are required to prevent formation of micronuclei and generation of DNA damage in cytokinesis with chromatin bridges.

The abscission checkpoint can also be activated by partial depletion of nucleoporin 153 (Nup153) and is evidenced by an accumulation of cells at the midbody stage that are unable to complete abscission^21^. Importantly, accumulation of cells at midbody stage after Nup153 depletion by Nup153 siRNA (siNup153) was prevented by co-transfection of cells with Clk1, Clk2 or Clk4 siRNA (Fig. 4g,h), whereas the frequency of cells in prometaphase was similar in all the above treatments (Supplementary Fig. 7b). Taken together, we propose that Clk1, Clk2 and Clk4 are required for the abscission checkpoint in response to chromatin bridges or partial depletion of Nup153.

### Clks localize to the midbody in an interdependent manner

**Figure 5:**
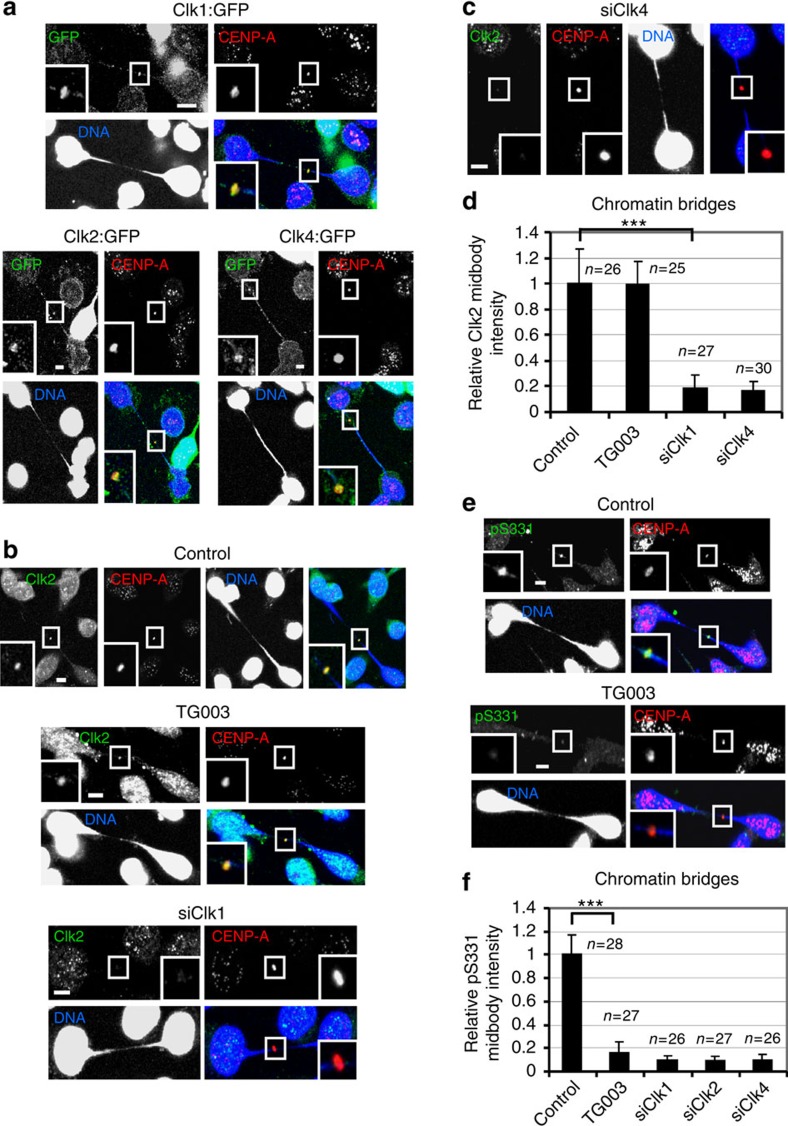
Clks localize to the midbody in a mutually dependent manner. (**a**) Cells expressing Clk1:GFP, Clk2:GFP or Clk4:GFP proteins were analysed in cytokinesis with chromatin bridges. Green, GFP; red, CENP-A; blue, DNA. A minimum of 22 cells from three independent experiments were examined per treatment. (**b**,**c**) Localization of Clk2. Cells were transfected with negative siRNA (control), Clk1 siRNA (siClk1), Clk4 siRNA (siClk4) or treated with TG003 for 5 h. Green, Clk2; red, CENP-A; blue, DNA. (**d**) Mean midbody intensity of Clk2 in cells treated as in **b**,**c**. (**e**) Localization of phosphorylated Aurora B–S331 (pS331) in cells treated as in **b**. Green, pS331; red, CENP-A; blue, DNA. Insets show magnified midbodies. Scale bars, 5 μm. (**f**) Mean midbody intensity of pS331 in cells transfected with Clk2 siRNA (siClk2) or treated as in **b**,**c**. Data are from *n* cells from three independent experiments. Error bars show s.d. Values in control were set to 1. ***P<0.001 compared with control. The Mann–Whitney *U*-test was used.

In control cells with chromatin bridges, endogenous Clk1, Clk2 or Clk4, or transiently expressed Clk1:GFP, Clk2:GFP or Clk4:GFP localized to the midbody where they co-localized with endogenous Aurora B (Fig. 5a,b and Supplementary Fig. 7c,d). Depletion of one Clk1, Clk2 or Clk4 protein by siRNA, but not inhibition of Clks by TG003, reduced localization of the other two Clks to the midbody by 86–95% compared with controls (Fig. 5b–d and Supplementary Fig. 8a–c), suggesting that all three Clk proteins stabilize each others presence in the midbody to impose the abscission checkpoint in response to chromatin bridges.

### Clks phosphorylate S331 in cytokinesis with DNA bridges

**Figure 6:**
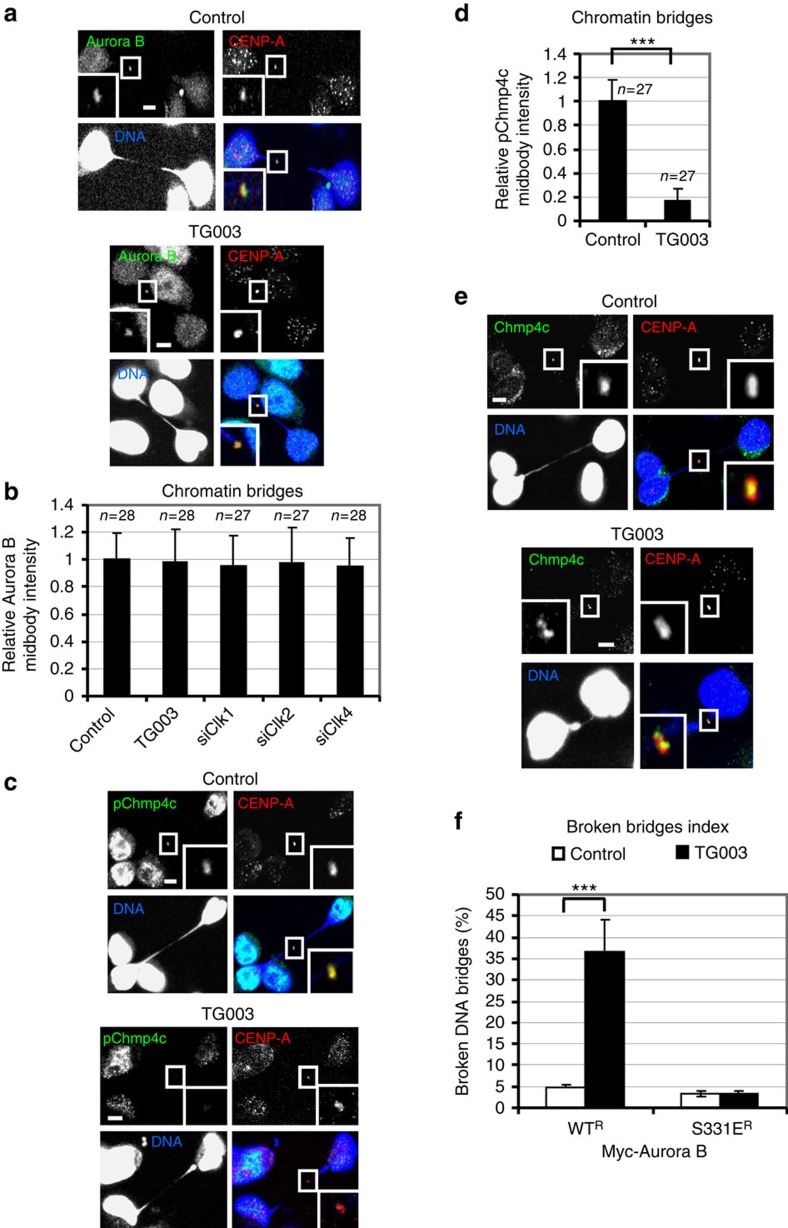
Clk inhibition reduces Chmp4c phosphorylation at the midbody in cells with chromatin bridges. (**a**) Localization of total Aurora B. Cells were transfected with negative siRNA (control) or treated with TG003 for 5 h. Green, Aurora B; red, CENP-A; blue, DNA. (**b**) Mean midbody intensity of Aurora B in cells treated as in **a** or transfected with Clk1 siRNA (siClk1), Clk2 siRNA (siClk2) or Clk4 siRNA (siClk4). (**c**) Localization of phosphorylated Chmp4c-S210, S214 and S215 (pChmp4c) in the absence (control) or presence of TG003 for 5 h. Green, pChmp4c; red, CENP-A; blue, DNA. (**d**) Mean midbody intensity of pChmp4c. Data are from *n* cells from three independent experiments. Error bars show s.d. Values in control were set to 1. ***P<0.001 compared with control. The Mann–Whitney *U*-test was used. (**e**) Localization of total Chmp4c in cells treated as in **c**. Green, Chmp4c; red, CENP-A; blue, DNA. Insets show magnified midbodies. Scale bars, 5 μm. (**f**) Broken bridges index analysis. Cells expressing siRNA-resistant forms of WT or S331E Myc-tagged Aurora B (Myc-Aurora B) were depleted of endogenous Aurora B by siRNA and treated as in **c**. Error bars show the s.d. from the mean from three independent experiments. A minimum of 50 cells in cytokinesis with chromatin bridges were analysed per experiment. ***P<0.001 compared with control. Student's *t*-test was used.

Control cells exhibited phosphorylated Aurora B–S331 at the midbody in late cytokinesis with DNA bridges (Fig. 5e). Inhibition of Clks by TG003 or depletion of Clk1, Clk2 or Clk4 by siRNA diminished Aurora B–S331 phosphorylation at the midbody compared with controls (Fig. 5e,f and Supplementary Fig. 8d). This was not due to impaired localization of total Aurora B at the midbody in Clk-deficient cells (Fig. 6a,b and Supplementary Fig. 9a).

We also examined localization of Mklp1 as a surrogate marker of Aurora B catalytic activity in late cytokinesis^24^. It was proposed that phosphorylation by Aurora B prevents premature entry of Mklp1 to the nucleus^24^. In control cells with chromatin bridges, Mklp1 did not localize to daughter nuclei, indicating active Aurora B (Supplementary Fig. 9b). In contrast, cells treated with TG003 or depleted of Aurora B by siRNA (as a positive control) exhibited Mklp1 inside the daughter nuclei and this was not due to premature abscission, because an intact intercellular bridge (as evidenced by α-tubulin staining) was visible in those cells (Supplementary Fig. 9b). We propose that Cdc-like kinase activity is required for Aurora B–S331 phosphorylation at the midbody and optimal Aurora B activation in cytokinesis with chromatin bridges.

### Clks regulate Chmp4c in cytokinesis with DNA bridges

Inhibition of Clks by TG003 reduced phosphorylation of Chmp4c-S210, S214 and S215 at the midbody compared with controls in cytokinesis with chromatin bridges (Fig. 6c,d). In addition, in control cells, total Chmp4c localized to the midbody as a single dot and co-localized with CENP-A (Fig. 6e). In contrast, in cells treated with TG003, Chmp4c localized to the midbody as two separate dots that were juxtaposed to CENP-A (Fig. 6e). These results show that Cdc-like kinase activity is required for optimal phosphorylation and localization of Chmp4c to the midbody in cytokinesis with DNA bridges.

### Aurora B–S331 phosphorylation prevents chromatin breakage

**Figure 7:**
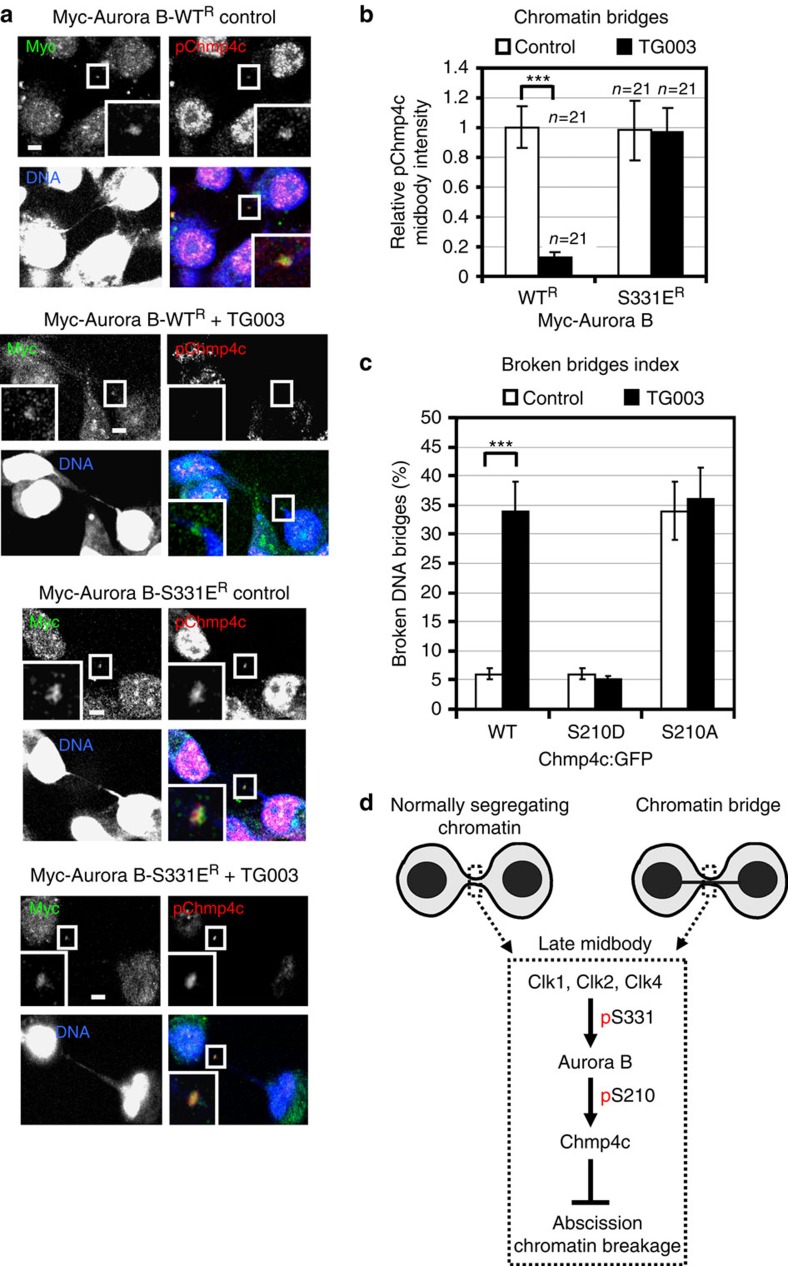
Expression of the phosphomimetic mutant S331E Aurora B rescues Chmp4c phosphorylation at the midbody in Clk-deficient cells. (**a**) Localization of phosphorylated Chmp4c-S210, S214 and S215 (pChmp4c). Cells expressing siRNA-resistant forms of WT or S331E Myc-tagged Aurora B (Myc-Aurora B) were depleted of endogenous Aurora B by siRNA in the absence (control) or presence of TG003 for 5 h. Green, Myc; red, pChmp4c; blue, DNA. Insets show magnified midbodies. Scale bars, 5 μm. (**b**) Mean midbody intensity of pChmp4c from cells treated as in **a**. Data are from *n* cells from three independent experiments. Values in control were set to 1. Error bars show s.d. ***P<0.001 compared with control. The Mann–Whitney *U*-test was used. (**c**) Broken bridges index analysis in cells expressing WT, S210D or S210A Chmp4c:GFP in the absence (control) or presence of TG003 for 5 h. Error bars show s.d. from the mean from three independent experiments. A minimum of 50 cells in cytokinesis with chromatin bridges were analysed per experiment. ***P<0.001 compared with control. Student's *t*-test was used. (**d**) Model for the role of Clks in abscission. p, phosphorylation.

In cells depleted of endogenous Aurora B by siRNA, expression of the phosphomimetic mutant S331E^R^, but not WT^R^, Myc-Aurora B prevented chromatin breakage after Clk inhibition by TG003 compared with controls (Fig. 6f). Furthermore, in control cells, WT^R^ or S331E^R^ Myc-Aurora B co-localized with phosphorylated Chmp4c to the midbody in late cytokinesis with chromatin bridges (Fig. 7a). Remarkably, expression of S331E^R^, but not WT^R^, Myc-Aurora B rescued phosphorylation of Chmp4c at the midbody after Clk inhibition compared with control cells (Fig. 7a,b). In addition, expression of the phosphomimetic mutant S210D, but not WT, Chmp4c:GFP prevented chromatin breakage and rescued localization of Chmp4c:GFP to the midbody as a single dot after TG003 treatment compared with control cells (Fig. 7c and Supplementary Fig. 9c). In contrast, expression of the non-phosphorylatable S210A Chmp4c:GFP mutant induced chromatin breakage and S210A Chmp4c localized to the midbody as two separate dots in the absence or the presence of the Clk inhibitor (Fig. 7c and Supplementary Fig. 9c). We propose that Clk-mediated Aurora B–S331 phosphorylation and subsequent Chmp4c-S210 phosphorylation by Aurora B are required to prevent chromatin breakage in late cytokinesis.

## Discussion

On the basis of those findings, we propose the following model for the role of Cdc-like kinases in abscission (Fig. 7d): Clk1, Clk2 and Clk4 localize to the midbody and phosphorylate Aurora B–S331 at midbody arms (early midbody) or midbody centre (late midbody), to induce Aurora B kinase activity. In turn, active Aurora B phosphorylates Chmp4c inside the Flemming body in several residues including S210, to delay abscission and prevent chromatin breakage in cytokinesis with chromatin bridges.

Our observation that reduced Aurora B kinase activity at the midbody after Clk inhibition leads to chromatin breakage in cells with chromatin bridges appears at odds with a previous study showing that when Aurora B is inhibited by a chemical inhibitor the cytokinetic bridge regresses and human cells become tetraploid^5^. As Aurora B exhibits relatively low levels of catalytic activity in the absence of S331 phosphorylation^10^, one possibility is that potent Aurora B inhibition by a chemical inhibitor may result in furrow regression, whereas low-level Aurora B kinase activity in Clk-deficient cells may be sufficient for stabilization of the cytokinetic bridge but may lead to premature abscission and DNA breakage. Therefore, the difference between the results of Steigemann *et al.*^5^ and our observations may be due to different degrees of Aurora B inhibition.

It has been proposed that local auto-activation through Aurora B clustering onto chromatin bridges can prevent DNA breakage by abscission in yeast^29^,^30^. Our model suggests a novel mechanism for activating the abscission checkpoint in human cells, through Clk-mediated Aurora B phosphorylation. This is in agreement with previous findings that Aurora B activity at intercellular canals does not exclusively depend on its auto-activation^5^. Our model also proposes a common pathway for activating an abscission delay in the absence or presence of trapped chromatin, consistent with a role for Aurora B in delaying abscission in normally segregating cells^5^. One possibility is that the abscission checkpoint is activated spontaneously, perhaps by monitoring midbody formation^4^, and that chromatin bridges sustain checkpoint signalling until chromosome segregation is completed, for example, through counteracting dephosphorylation of Aurora B by inhibitory phosphatases. In conclusion, our study describes the identification of a potential novel component of the abscission checkpoint acting upstream of Aurora B.

## Methods

### Antibodies and plasmids

Monoclonal antibody against Myc (9E10), rabbit polyclonal antibodies against GFP (FL), Mklp1 (N-19) and Nup153 (H-161), and a goat polyclonal antibody against Clk1 (N-17) were obtained from Santa Cruz Biotechnology. Antibodies against normal rabbit (sc-2027) or goat IgG (sc-2028) used in immunoprecipitations were also from Santa Cruz Biotechnology. Rabbit polyclonal anti-Clk2 (ab86147), anti-Clk4 (ab104321), anti-Chmp4c (ab155668) and anti-Aurora B (ab2254; used in immunofluorescence and in Fig. 2g) antibodies were from Abcam. Mouse monoclonal antibodies against α-tubulin (DM1A) and actin (AC-40) were from Sigma, anti-CENP-A (3-19) was from GeneTex, anti-AIM1 (Aurora B; used in western blotting and in Fig. 1h) was from BD Biosciences, anti-lamin B2 (E-3) was from Life Technologies and anti-phospho-Histone H2A.X (S139; clone JBW301; γ-H2AX) was from Millipore. Anti-pS331 rabbit polyclonal antiserum against phosphorylated S331 of human Aurora-B was previously described^10^. Anti-pChmp4c rabbit polyclonal antiserum against phosophorylated S210, S214 and S215 of human Chmp4c was a gift from Pier Paolo D'Avino (University of Cambridge, Cambridge, UK)^7^. Antibody dilutions were 1:100 for immunofluorescence and 1:2,000 for western blotting.

Plasmids pCR3.1 GFP-EXN/chmp4c and pCR3.1 GFP-EXN/chmp4b encoding human Chmp4c or Chmp4b fused to GFP, respectively, were from Paul Beniasz (The Aaron Diamond AIDS Research Center, New York, USA)^31^. pCMV-GFP::Chmp4a encoding human Chmp4a fused to GFP was from Pier Paolo D'Avino (University of Cambridge)^7^. pEGFP/clk2 encoding human Clk2 fused to GFP in pEGFP-C2 vector (Clontech) was from Axel Ullrich (Max-Planck Institute for Biochemistry, Martinsried, Germany)^14^. pEGFP/clk1 and pEGFP/clk4 encoding human Clk1 or Clk4 fused to GFP, respectively, into pEGFP-C1 vector (Clontech) were from John Bell (Ottawa Hospital Research Institute, Ontario, Canada). Plasmid mCh-α-tubulin encoding mCherry-tagged α-tubulin was a gift from Gia Voeltz (Addgene plasmid 49149)^32^. Plasmid pEGFP-vps4-K173Q encoding human Vps4 harbouring the K173Q point mutation fused to enhanced green fluorescent protein (EGFP) into pEGFP-C1 vector (Clontech) was a gift from Wesley Sundquist (University of Utah, Salt Lake City, USA)^9^ and plasmid pEGFP-N1 coding for GFP under cytomegalovirus promoter was obtained from Takara Bio Inc.

### siRNA sequences

Negative siRNA (a pool of four different siRNAs: 5′-UAAGGCUAUGAAGAGAUAC-3′, 5′-AUGUAUUGGCCUGUAUUAG-3′, 5′-AUGAACGUGAAUUGCUCAA-3′, 5′-UGGUUUACAUGUCGACUAA-3′) and human Aurora B siRNA (5′-CCAAACUGCUCAGGCAUAA-3′) were from Thermo Scientific. Human Clk1 (5′-GUAAACCUCUGAAGGAAUU-3′), Clk2 (5′-CCUUCGAUUUCCUCAAAGA-3′), Clk4 (5′-GCAAACCGUUGAAGGAAUU-3′), Clk1b (5′-GUAGACAUGUAGCAGUAAA-3′), Clk2b (5′-CAGCUCGACUUGAGAUCAA-3′), Clk4b (5′-GGAAAGGCAUGCAGUUUGU-3′), Chmp4c (a pool of three different siRNAs: 5′-GCUUGGGCUACCUAAACUA-3′, 5′-GUCAUGUGCAUACAUAGAA-3′, 5′-GUAGAGGAGUCUUAUAUGA-3′), CENP-A (a pool of three different siRNAs: 5′-GCAUGACUUUCCUCUGUAA-3′, 5′-CUAGUAAAUUCCUGUCAAA-3′, 5′-GUAUCAUAACAGUUCAGAA-3′) and Nup153 (5′-AAGGCAGACUCUACCAAAUGUUU-3′) siRNAs were from Santa Cruz Biotechnology. Only the sense sequences of the siRNA duplexes are shown and all sequences are provided in 5′→3′ orientation.

### Recombinant and purified proteins

Recombinant Clk1, Clk2 and Clk4 proteins were from EMD Millipore and KD Aurora B^KD^ (D200A) was from Upstate Biotechnology. WT or T1052G (changing S331 to alanine) human Aurora B complementary DNAs into pET-28a(+) vectors (EMD) were expressed in BL21 (DE3) cells (Agilent Technologies). Proteins were purified using the 6 × His purification kit (B-PER; Thermo Fisher Scientific) and used as substrates in kinase reactions. Human GST-tagged Aurora B protein was expressed in BL21 (DE3) cells and purified using glutathione-agarose beads (Santa Cruz Biotechnology).

### Mutagenesis and cloning

Point mutations were generated using the QuikChange site-directed mutagenesis kit (Agilent Technologies). To generate siRNA-resistant forms of Myc-Aurora B, the Myc/Aurora B plasmid coding 6 × Myc-tagged human Aurora B in pcDNA5/FRT/TO vector (Invitrogen)^10^ was used to introduce C919T, G921C and C922T point mutations giving resistance to the Aurora B siRNA. This plasmid was then used to make T991G, C992A and T993G point mutations changing Aurora B–S331 to glutamic acid, or T1052G point mutation changing Aurora B–S331 to alanine. pCR3.1 GFP-EXN/chmp4c was used to generate T628G point mutation changing Chmp4c-S210 to alanine or T628G and C629A point mutations changing Chmp4c-S210 to aspartic acid.

To generate GST–Aurora B, human 6 × Myc-Aurora B cDNA in pcDNA3 vector (Invitrogen)^10^ was excised with EcoRI–NotI and introduced into the pGEX4T-1 plasmid (GE Healthcare).

### Cell culture and treatments

Human colon carcinoma BE cells (a gift from Simon Wilkinson and Christopher Marshall, Institute of Cancer Research, London, UK) and cervical carcinoma HeLa cells stably expressing LAP2b fused to RFP (a kind gift from Daniel Gerlich, Institute of Molecular Biotechnology, Vienna, Austria)^5^ were grown in DMEM medium (Gibco) containing 10% fetal bovine serum at 37 °C in 5% CO_2_. BE cells stably expressing H2B fused to GFP were as described^11^. Cells were treated with 1 μM TG003 (Sigma) or 300 nM UCN-01 (Sigma) for 5 h before analysis, unless otherwise stated. Negative siRNA or siRNA duplexes designed to repress human Aurora B, Chk2, Clk1, Clk2, Clk4, Chmp4c, CENP-A or Nup153 were transfected into BE cells 24 h before analysis using Lipofectamine 2000 (Invitrogen). For expression of GFP proteins, plasmids were transfected into cells in the absence or presence of appropriate siRNA duplexes 24 h before analysis or further drug treatment using Turbofect (Life Technologies).

### Enrichment of cells in cytokinesis

BE cells were treated with 50 ng ml^−1^ nocodazole (Sigma) for 16 h, washed twice with PBS and released in fresh medium for 2 h. Microscopic examination had shown ∼33% of the cells were at midbody stage after this treatment.

### Time-lapse imaging

BE cells stably expressing H2B:GFP or HeLa cells stably expressing LAP2b:RFP were seeded onto Petri dishes with a 30-mm glass base (IWAKI; Asahi glass Co., Ltd). Phase-contrast and fluorescence images were taken every 5 min using an inverted fluorescence microscope (DMIRE2; Leica) and a × 40 Plan Neo 0.6 NA Ph2 objective. Imaging was performed in air, at 37 °C in 5% CO_2_, using a camera (DFC300FX; Leica) and IM50 acquisition software (Leica). To visualize mCherry:tubulin, BE cells were transfected with the mCh-α-tubulin plasmid, transferred onto glass base Petri dishes and analysed 24 h after transfection.

### Indirect immufluorescence microscopy

For phospho-Aurora B–S331 or phospho-Chmp4c-S210, S214 and S215 staining, cells were extracted in pre-warmed (37 °C) Phem buffer (60 mM PIPES, 25 mM HEPES pH 7.0, 10 mM EGTA and 4 mM MgSO_4_) supplemented with 0.5% CHAPS and 100 nM microcystin (Sigma-Aldrich) for 5 min at room temperature, fixed with pre-warmed (37 °C) 4% paraformaldehyde in Phem buffer for 10 min at room temperature, permeabilized in 0.5% Triton X-100 in Phem buffer for 2 min at room temperature, washed twice with PBS and immunostained as appropriate. For Clk1, Clk2 or Clk4 staining, cells were fixed in ice-cold methanol for 5 min at −20 °C, washed twice with PBS at room temperature and immunostained.

For all other fluorescence microscopy applications, cells were fixed in 4% paraformaldehyde in cytoskeleton buffer (1.1 M Na_2_HPO_4_, 0.4 M KH_2_PO_4_, 137 mM NaCl, 5 mM KCl, 2 mM MgCl_2_, 2 mM EGTA, 5 mM PIPES and 5 mM glucose pH 6.1) for 5 min at 37 °C, permeabilized in 0.5% Triton X-100 in cytoskeleton buffer, washed twice with PBS at room temperature and immunostained^12^.

Fluorescein isothiocyanate- or rhodamine-TRITC-conjugated secondary antibodies (Jackson ImmunoResearch Laboratories, Inc.) were used as appropriate. Actin was visualized with Fluorescein–Phalloidin (Life Technologies) or TRITC–Phalloidin (Millipore). DNA was stained with 10 μM DRAQ7 (644/678 nm; Abcam) and cells were mounted in Vectashield medium (Vector Laboratories). Images were collected using a laser-scanning spectral confocal microscope (TCS SP2; Leica), LCS Lite software (Leica) and a × 63 Apochromat 1.40 numerical aperture oil objective. The low fluorescence immersion oil (11513859; Leica) was used and imaging was performed at room temperature. Average projections of image stacks were obtained using the LCS Lite software.

Fluorescence intensity signals at midbodies were quantified using the LCS Lite ellipse tool by analysing an image area of 2 μm^2^ around each midbody and intensity values were normalized versus values obtained by analysing an identical area within the cell immediately adjacent on the midbody^33^. A minimum of seven cells per experiment from three independent experiments were analyzed for each treatment (that is, *n* is at least 21 cells per treatment) and s.d. from *n* cells was calculated. To analyse midbody thickness, the diameter of each microtubule bundle at the midbody was measured using the LCS Lite line tool and the average value calculated.

### Midbody purification

After enrichment in cytokinesis, cells were centrifuged at 200 *g* for 3 min and gently resuspended in 100 volumes hypotonic swelling solution (1 mM PIPES pH 7, 1 mM MgCl_2_, 5 mg ml^−1^ taxol (Applichem), 5 μg ml^−1^ leupeptin, 50 μg ml^−1^ phenylmethylsulfonyl fluoride (PMSF) and 5 μg ml^−1^ aprotinin)^7^. Cells were centrifuged at 200 *g* for 3 min, resuspended in a lysis solution containing 1 mM PIPES pH 7, 1 mM EGTA, 1% NP40, 5 mg ml^−1^ taxol, 5 μg ml^−1^ leupeptin, 50 μg ml^−1^ PMSF and 5 μg ml^−1^ aprotinin, and vortexed vigorously. After addition of 0.3 volumes of cold 50 mM 2-(*N*-morpholino) ethane sulphonic acid (MES) pH 6.3, cells were incubated on ice for 20 min and then centrifuged at 250 *g* for 10 min. The supernatant was then transferred to a new tube and centrifuged at 740 *g* for 20 min, to pellet midbodies. The pellet was resuspended in 50 mM MES pH 6.3, layered over a cushion of 40% glycerol in 50 mM MES pH 6.3 and centrifuged at 2,800 *g* for 45 min. Midbodies were resuspended in MES, plated on poly-lysine-coated coverslips and processed for immunofluorescence microscopy.

### *In vitro* kinase assays

For Clk1, Clk2 or Clk4 *in vitro* kinase assays, 0.5 μg recombinant Clk1, Clk2 or Clk4 was incubated with 0.5–1 μg protein substrate in 20 μl kinase buffer (40 mM MOPS-ΝαOΗ pH 7.0, 1 mM EDTA, 100 μM ATP and 2 μCi γ-ATP) for 30 min at 30 °C before analysis by SDS–PAGE and autoradiography.

### GST pulldowns and immunoprecipitations

Cells were sonicated 3− for 10 s in ice-cold immunoprecipitation buffer (50 mM HEPES pH 7.5, 150 mM NaCl, 1 mM EDTA, 2.5 mM EGTA, 0.1% Tween 20, 10% glycerol, 0.1 mM PMSF, 10 mM sodium β-glycerophosphate, 0.1 mM Na_3_VO_4_, 1 mM NaF, 10 μg ml^−1^ leupeptin and 10 μg ml^−1^ aprotinin). For GST pull-down assays, 1 mg cell lysate was incubated with 10 μg agarose-bound GST proteins for 6 h at 4 °C. For immunoprecipitations, 3–4 mg cell lysate was incubated with 0.5 μg anti-Clk or anti-IgG antibody for 16 h followed by addition of 20 μl protein A/G PLUS–agarose beads (Santa Cruz Biotechnology) for 1 h at 4 °C. Samples were spun down, washed four times with immunoprecipitation buffer and analysed by SDS–PAGE.

### Western blotting

Cells were lysed in ice-cold whole-cell extract buffer (20 mM HEPES, 5 mM EDTA, 10 mM EGTA, 0.4 M KCl, 0.4% Triton X-100, 10% glycerol, 5 mM NaF, 1 mM dithiothreitol, 5 μg ml^−1^ leupeptin, 50 μg ml^−1^ PMSF, 1 mM benzamidine, 5 μg ml^−1^ aprotinin and 1 mM Na_3_VO_4_) for 30 min on ice. Lysates were cleared by centrifugation at 15,000 *g* for 10 min, analysed by SDS–PAGE and transferred onto nitrocellulose membrane (Santa Cruz Biotechnology).

For Nup153 and lamin B2 staining, nuclear extracts were prepared using the NE-PER Nuclear and Cytoplasmic Extraction Reagents kit (Life Technologies) following the manufacturer's protocol and analysed by SDS–PAGE.

## Additional information

**How to cite this article:** Petsalaki, E. & Zachos. G Clks 1, 2 and 4 prevent chromatin breakage by regulating the Aurora B-dependent abscission checkpoint. *Nat. Commun.* 7:11451 doi: 10.1038/ncomms11451 (2016).

## Supplementary Material

Supplementary Figures Supplementary Figures 1-9

Supplementary Movie 1 Midbody disassembly in control cells. BE cells transiently expressing mCherry:tubulin (red) were analysed by time-lapse fluorescence microscopy. Frames were taken every 5 min for 40 min. Time counters show min:sec.

Supplementary Movie 2 Midbody disassembly in control cells. BE cells transiently expressing mCherry:tubulin (red) were analysed by phase contrast in cytokinesis. Frames were taken every 5 min for 40 min. Time counters show min:sec.

Supplementary Movie 3 Midbody disassembly in Clk-deficient cells. BE cells transiently expressing mCherry:tubulin (red) were treated with TG003 and analysed by time-lapse fluorescence microscopy. Frames were taken every 5 min for 25 min. Time counters show min:sec.

Supplementary Movie 4 Midbody disassembly in Clk-deficient cells. BE cells transiently expressing mCherry:tubulin (red) were treated with TG003 and analysed by phase contrast in cytokinesis. Frames were taken every 5 min for 25 min. Time counters show min:sec.

Supplementary Movie 5 Control cells exhibit stable LAP2b intercellular bridges in cytokinesis. HeLa cells stably expressing LAP2b:RFP (red) were analysed by time-lapse fluorescence microscopy. Frames were taken every 5 min for 60 min. Time counter shows min:sec.

Supplementary Movie 6 Breakage of LAP2b intercellular bridges in Clk-deficient cells. HeLa cells stably expressing LAP2b:RFP (red) were treated with TG003 and analysed by time-lapse fluorescence microscopy. Frames were taken every 5 min for 20 min. Time counters show min:sec.

Supplementary Movie 7 Breakage of LAP2b intercellular bridges in Clk-deficient cells. HeLa cells stably expressing LAP2b:RFP (red) were treated with TG003 and analysed by time-lapse fluorescence microscopy. Frames were taken every 5 min for 25 min. Time counters show min:sec.
