# Supplementary material for: Clks 1, 2 and 4 prevent chromatin breakage by regulating the Aurora B-dependent abscission checkpoint
Source: Nat Commun. 2016 Apr 29;7:11451. doi: 10.1038/ncomms11451 (PMC4855533; doi:10.1038/ncomms11451)
Supplement: Supplementary Figures — 1-9 [file ncomms11451-s1.pdf]

Supplementary Information

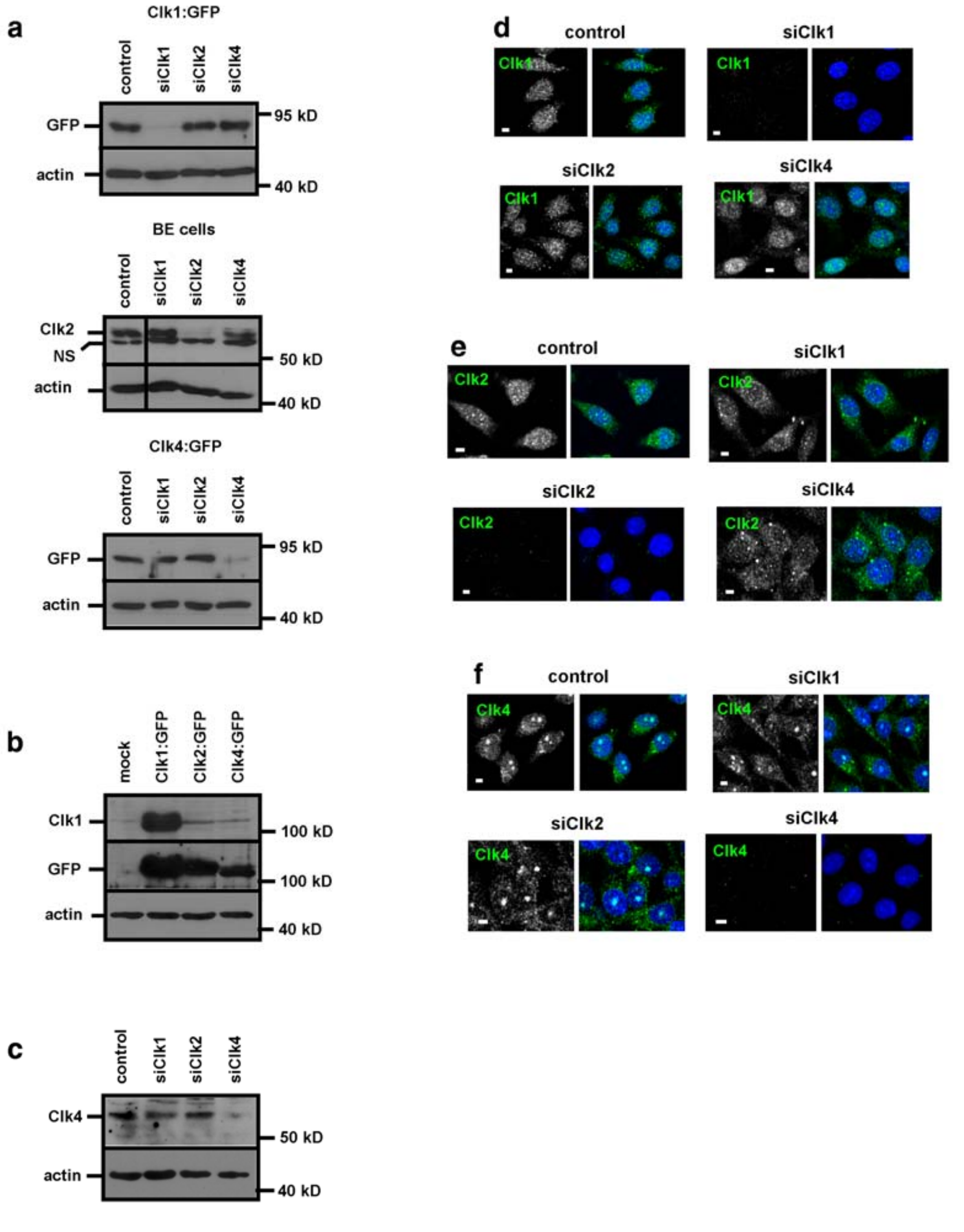

Supplementary Figure 1. Validation of Clk antibodies and Clk siRNAs. (a) Clk1, Clk2 and Clk4 siRNAs do not cross-react. Western blot analysis of total GFP, endogenous Clk2 and actin in BE cells or cells expressing Clk1:GFP or Clk4:GFP, after transfection with negative siRNA (control), Clk1 siRNA (siClk1), Clk2 siRNA

(siClk2) or Clk4 siRNA (siClk4). NS, non specific. **(b)** The Clk1 antibody detects transiently expressed Clk1, but not Clk2 or Clk4. Western blot analysis of total Clk1, GFP and actin in mock-transfected cells (mock) or cells expressing Clk1:GFP, Clk2:GFP or Clk4:GFP. **(c)** The Clk4 antibody does not cross-react with endogenous Clk1 or Clk2. Western blot analysis of total Clk4 and actin in cells transfected with negative siRNA (control), Clk1 siRNA (siClk1), Clk2 siRNA (siClk2) or Clk4 siRNA (siClk4). The Clk4 signal is only reduced in cells transfected with siClk4. **(d-f)** Specificity of endogenous Clk1 **(d)**, Clk2 **(e)** or Clk4 **(f)** staining by immunofluorescence. The Clk1 signal is only reduced in cells transfected with Clk1 siRNA, Clk2 staining is only reduced in cells transfected with Clk2 siRNA and the Clk4 signal is only reduced in cells transfected with Clk4 siRNA compared to controls. Green, Clk1, Clk2 or Clk4; Blue, DNA. Bars, 5 μm.

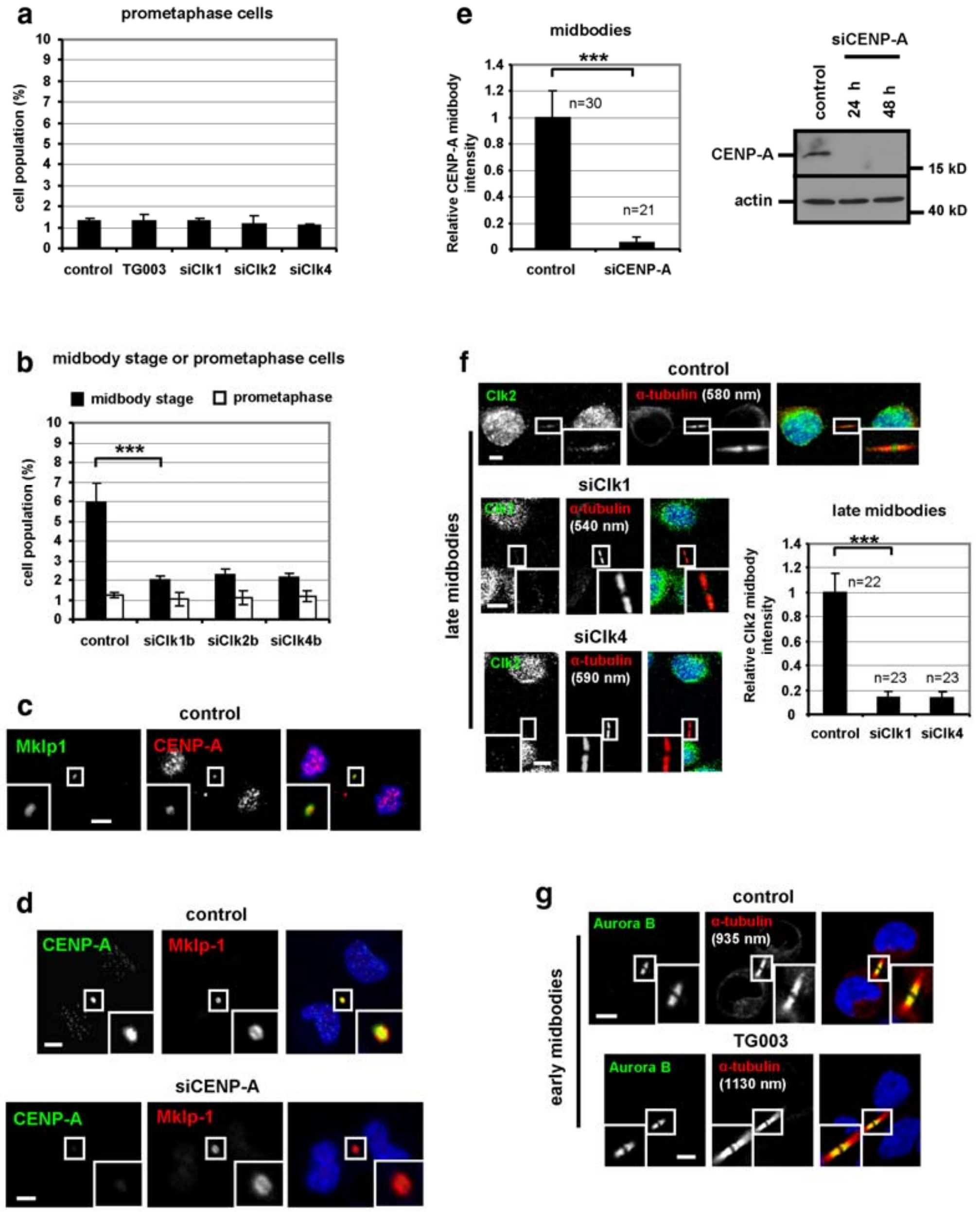

Supplementary Figure 2. Validation of CENP-A as a midbody marker. (a, b) Frequency of prometaphase or midbody stage cells. Cells were transfected with negative siRNA (control), Clk1 siRNA (siClk1 or siClk1b), Clk2 siRNA (siClk2 or siClk2b), Clk4 siRNA (siClk4 or siClk4b) or treated with TG003 for 5 h. Error bars show the standard deviation from the mean from three independent experiments. A

minimum of 300 cells (for midbody stage) or 800 cells (for prometaphase) were analyzed per experiment. Three asterisks, $p<0.001$ compared with control. The Student's t-test was used. **(c)** Endogenous CENP-A colocalises with Mklp1 at the midbody. Green, Mklp1; red, CENP-A; Blue, DNA. **(d)** Depletion of CENP-A diminishes CENP-A staining at the midbody. Cells were transfected with negative siRNA (control) or CENP-A siRNA (siCENP-A). Green, CENP-A; red, Mklp1; Blue, DNA. **(e)** Mean midbody intensity of CENP-A (left) and Western blot analysis of total CENP-A and actin (right) in cells transfected as in **d**. **(f)** Localisation and mean midbody intensity of Clk2 in cells treated as in **a**. Green, Clk2; red, α-tubulin; Blue, DNA. Data is from *n* cells from three independent experiments. Values in control were set to 1. Three asterisks, $p<0.001$ compared with control. The Mann-Whitney U test was used. **(g)** Localisation of Aurora B in the absence (control) or presence of TG003 for 5 h. Green, Aurora B; red, α-tubulin; Blue, DNA. 30 cells from three independent experiments were examined per treatment. Tubulin values indicate midbody thickness. Insets show magnified midbodies. Bars, 5 μm.

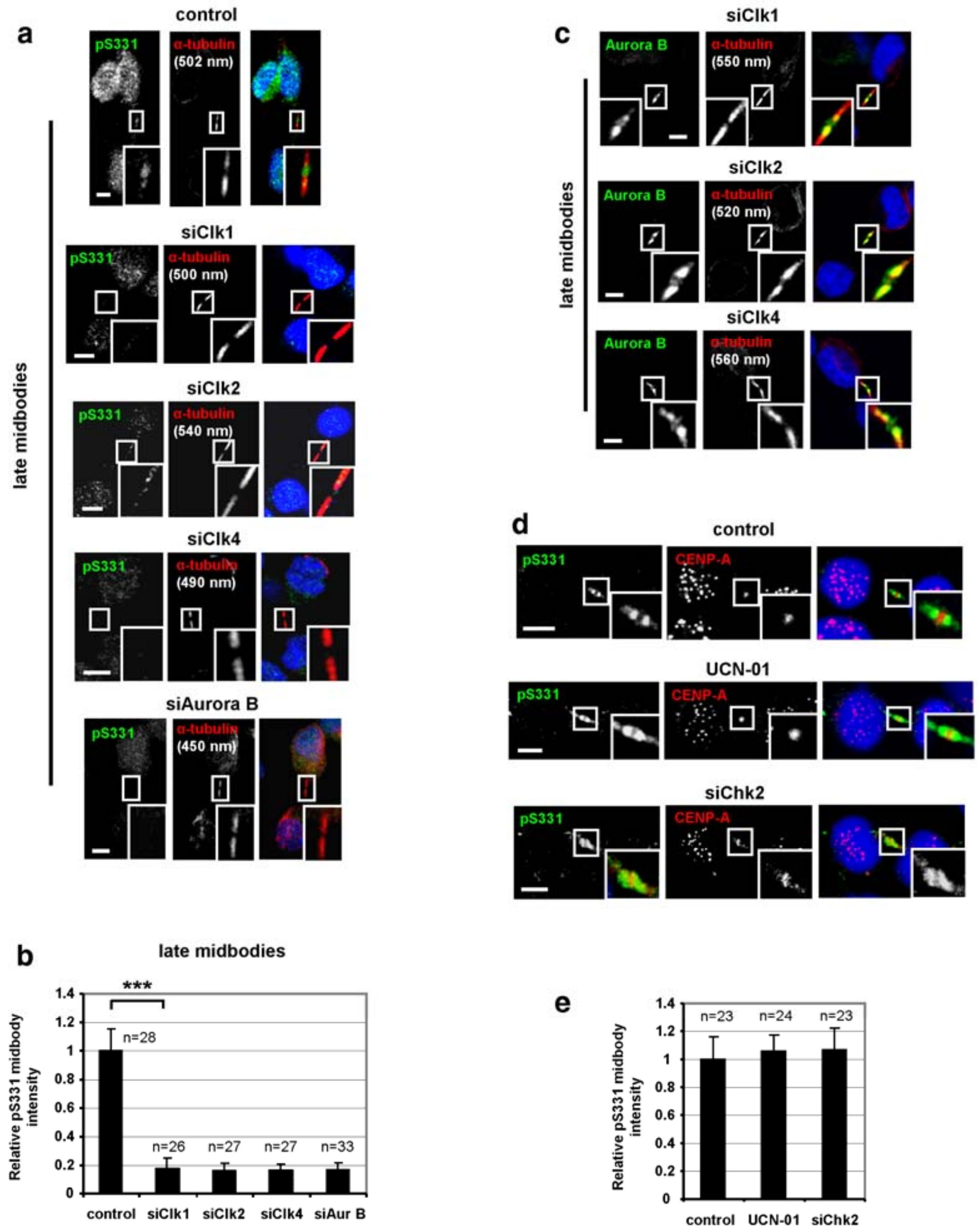

Supplementary Figure 3. Depletion of Clks, but not Chk1 inhibition or Chk2-depletion, reduces Aurora B-S331 phosphorylation at the midbody. (a) Localisation of phosphorylated Aurora B-S331 (pS331) in cells transfected with negative siRNA (control), Clk1 siRNA (siClk1), Clk2 siRNA (siClk2), Clk4 siRNA (siClk4) or Aurora B siRNA (siAurora B). Green, pS331; red, α-tubulin; Blue, DNA.

(b) Mean midbody intensity of pS331 in cells transfected as in **a**. Data is from *n* cells from three independent experiments. Values in control were set to 1. Three asterisks, $p<0.001$ compared with control. The Mann-Whitney U test was used. siAur B, siAurora B. **(c)** Localisation of total Aurora B in cells transfected as in **a**. Green, Aurora B; red, α-tubulin; Blue, DNA. Tubulin values indicate midbody thickness. **(d)** Localisation of phospho-Aurora B-S331 (pS331) in cells transfected with negative siRNA (control), Chk2 siRNA (siChk2), or treated with UCN-01 for 5 h. Green, pS331; red, CENP-A; Blue, DNA. Insets show magnified midbodies. Bars, 5 μm. **(e)** Mean midbody intensity of pS331 in cells treated as in **d**. Data is from *n* cells from three independent experiments. Values in control were set to 1. Error bars show standard deviation.

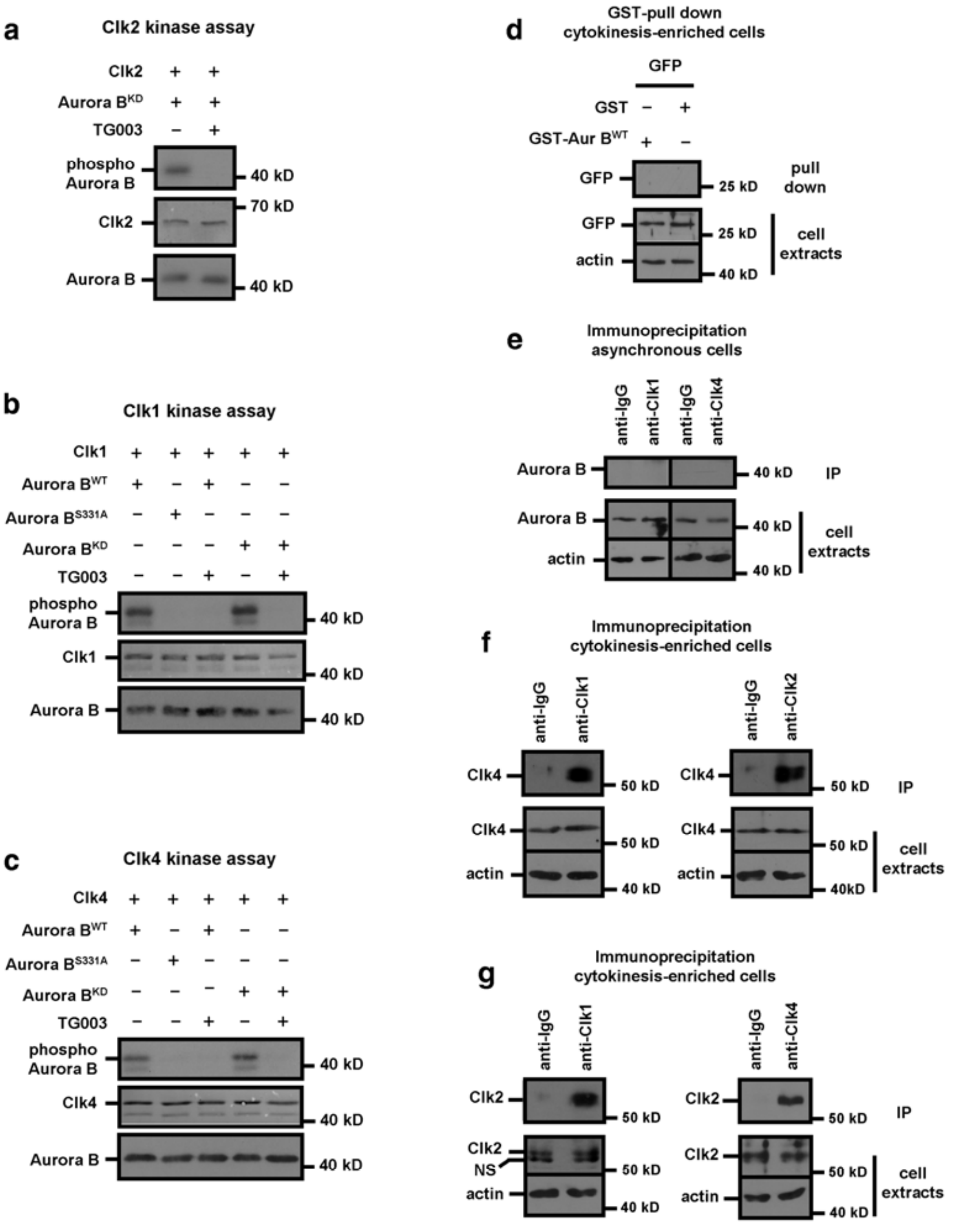

Supplementary Figure 4. Clk1, Clk2 and Clk4 phosphorylate Aurora B *in vitro*.
(a-c) *In vitro* kinase assays. Autoradiography analysis of Aurora B substrates (top), Ponceau staining of Clk kinases (middle) and western blot analysis of Aurora B substrates (bottom). **(d)** GFP does associate with GST-Aurora B or GST. Lysates from cytokinesis-enriched cells expressing GFP were incubated with 10 μg

glutathione–agarose-bound wild-type (WT) GST–tagged Aurora B (GST-Aur B^{WT}) or GST. Associated GFP (top) or total GFP and actin (bottom) were detected by western blotting. **(e)** Immunoprecipitation assay from asynchronous cells. Immunoprecipitated (IP) Aurora B (top) and total Aurora B and actin (bottom) were detected by western blotting. **(f, g)** Immunoprecipitation assays from cells enriched in cytokinesis. Immunoprecipitated (IP) Clk4 or Clk2 (top) and total Clk4, Clk2 and actin (bottom) were detected by western blotting. NS, non specific.

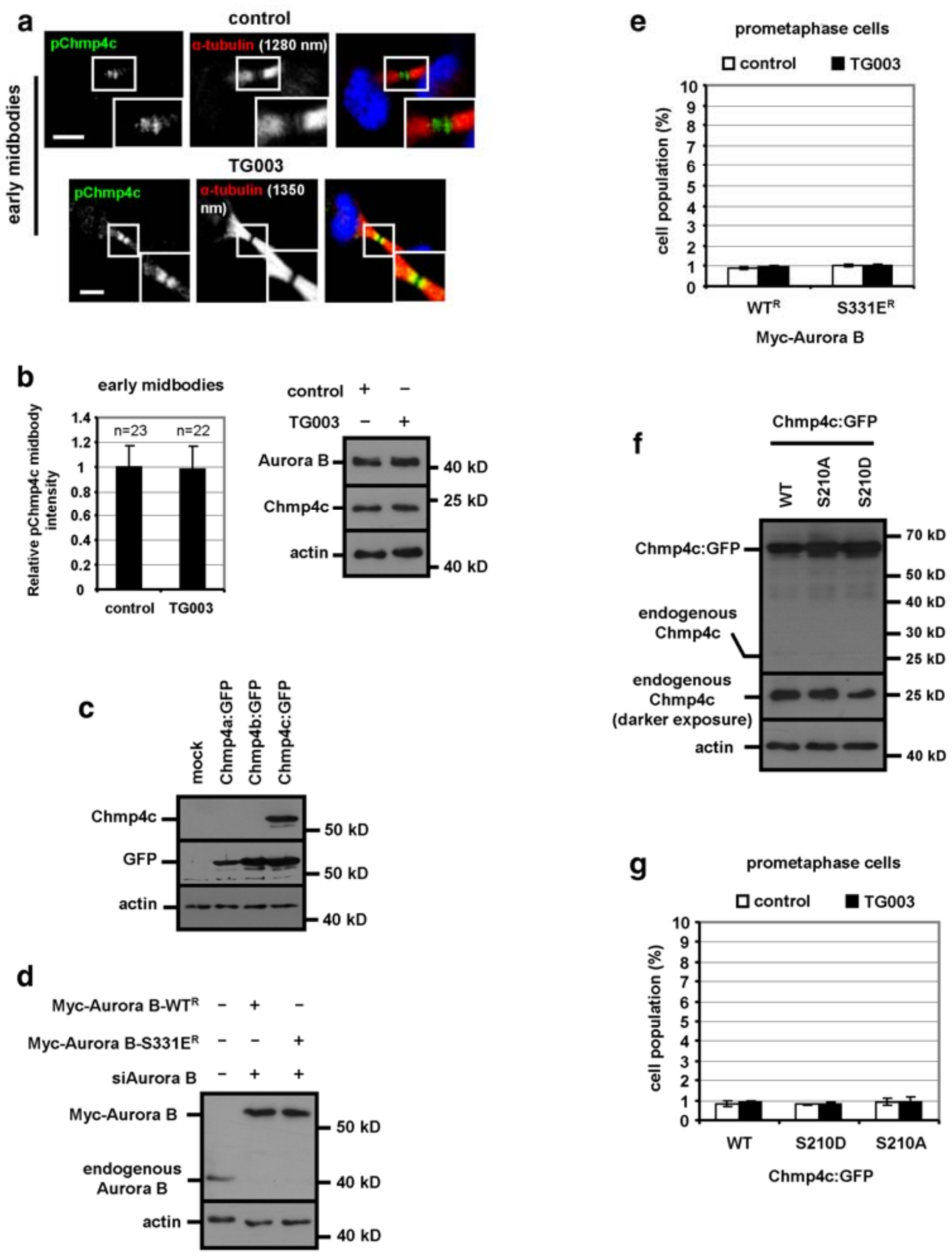

Supplementary Figure 5. Clk inhibition does not reduce Chmp4c phosphorylation in early midbodies. (a) Localisation of phosphorylated Chmp4c-S210, S214, S215 (pChmp4c) in early midbodies in the absence (control) or presence of TG003 for 5 h. Green, pChmp4c; red, α-tubulin; Blue, DNA. Tubulin values indicate midbody thickness. Insets show magnified midbodies. Bars, 5 μm. **(b)** Mean

midbody intensity of pChmp4c (left) and Western blot analysis of total Aurora B, Chmp4c and actin (right) in cells treated as in **a**. Data is from *n* cells from three independent experiments. Values in control were set to 1. Error bars show standard deviation. **(c)** The total Chmp4c antibody used detects transiently expressed Chmp4c, but not Chmp4a or Chmp4b. Western blot analysis of total Chmp4c, GFP and actin in mock-transfected cells (mock) or cells expressing Chmp4a:GFP, Chmp4b:GFP or Chmp4c:GFP. **(d)** Western blot analysis of total Aurora B and actin in cells expressing siRNA-resistant forms of WT or S331E Myc-tagged Aurora B (Myc-Aurora B) after transfection with Aurora B siRNA. **(e)** Frequency of prometaphase cells in cells transfected as in **d**, in the absence (control) or presence of TG003 for 5h. **(f)** Western blot analysis of total Chmp4c and actin in cells expressing WT, S210A or S210D Chmp4c:GFP. **(g)** Frequency of prometaphase cells in cells transfected as in **f**, in the absence (control) or presence of TG003 for 5h. Error bars show the standard deviation from the mean from three independent experiments. A minimum of 800 cells were analyzed per experiment.

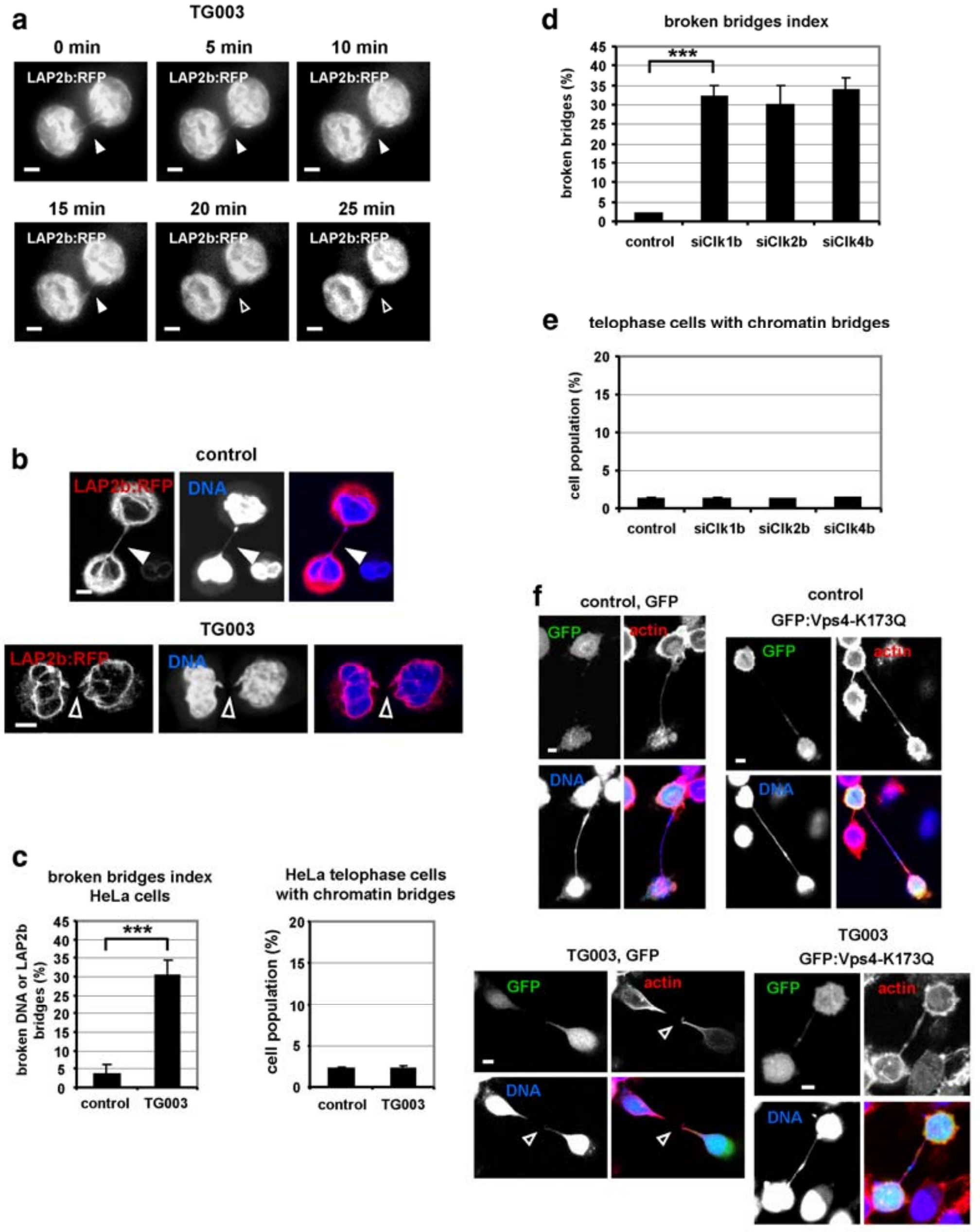

Supplementary Figure 6. Clk inhibition promotes breakage of LAP2b or chromatin bridges. (a) HeLa cells expressing LAP2b:RFP were analysed by time-lapse microscopy in the presence of TG003. Time from formation of the LAP2b:RFP bridge is indicated. **(b)** Intercellular bridges in fixed HeLa cells in the absence (control) or presence of TG003 for 5 h. Red, LAP2b:RFP; Blue, DNA. **(c)** Broken

bridges index analysis (left) and frequency of telophase cells with chromatin bridges (both intact and broken; right) in HeLa cells treated as in **b**. Error bars show the standard deviation from the mean from three independent experiments. A minimum of 50 cells with chromatin bridges (left) or 300 cells (right) were analyzed per experiment. **(d)** Broken bridges index analysis in BE cells transfected with negative siRNA (control), Clk1 siRNA (siClk1b), Clk2 siRNA (siClk2b) or Clk4 siRNA (siClk4b). Error bars show the standard deviation from the mean from three independent experiments. A minimum of 50 cells with chromatin bridges were analyzed per experiment. Three asterisks, $p<0.001$ compared with control. The Student's t-test was used. **(e)** Frequency of telophase cells with chromatin bridges (both intact and broken) in cells treated as in **d**. A minimum of 300 cells were analyzed per experiment. **(f)** Cells expressing GFP or GFP:Vps4-K173Q were treated as in **b**. Green, GFP ; red, actin; Blue, DNA. Intact intercellular or chromatin bridges are indicated by solid arrowheads and broken bridges by open arrowheads. Bars, 5 μm.

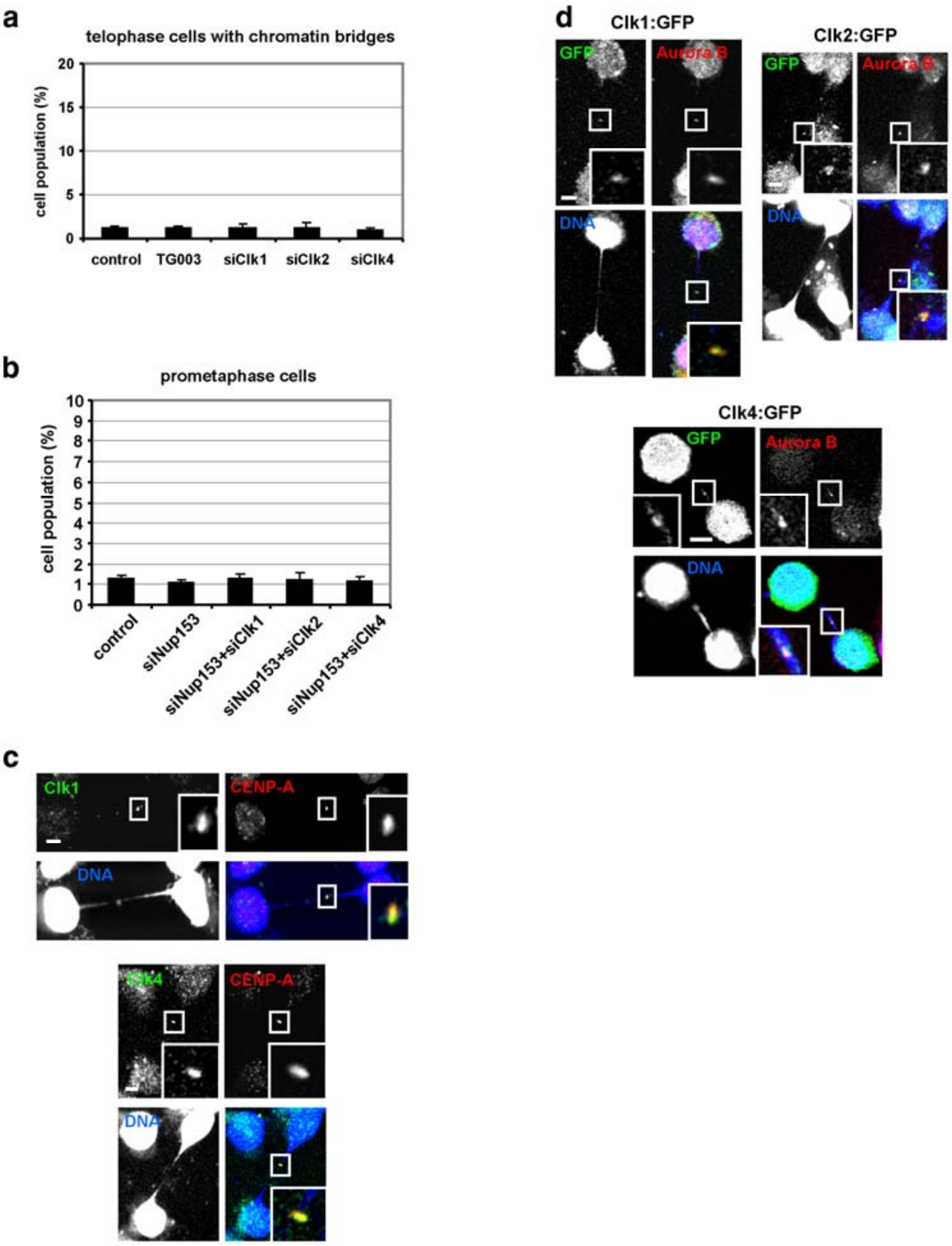

Supplementary Figure 7. Clk1, Clk2 or Clk4 colocalise with Aurora B at the midbody in cytokinesis with chromatin bridges. (a) Frequency of telophase cells with chromatin bridges (both intact and broken). Cells were transfected with negative siRNA (control), Clk1 siRNA (siClk1), Clk2 siRNA (siClk2), Clk4 siRNA (siClk4) or treated with TG003 for 5 h. A minimum of 300 cells were analyzed per

experiment. **(b)** Frequency of prometaphase cells. Cells were transfected with negative siRNA (control), Nup153 siRNA (siNup153), or combinations of Nup153 siRNA and Clk1 siRNA (siNup153+siClk1), Nup153 siRNA and Clk2 siRNA (siNup153+siClk2), or Nup153 siRNA and Clk4 siRNA (siNup153+siClk4). A minimum of 800 cells were analyzed per experiment. **(c)** Localisation of endogenous Clk1 or Clk4. Green, Clk1 or Clk4; red, CENP-A; Blue, DNA. 30 cells from three independent experiments were examined. **(d)** Cells expressing Clk1:GFP, Clk2:GFP or Clk4:GFP were analysed. Green, GFP; red, Aurora B; Blue, DNA. 22 cells from three independent experiments were examined.

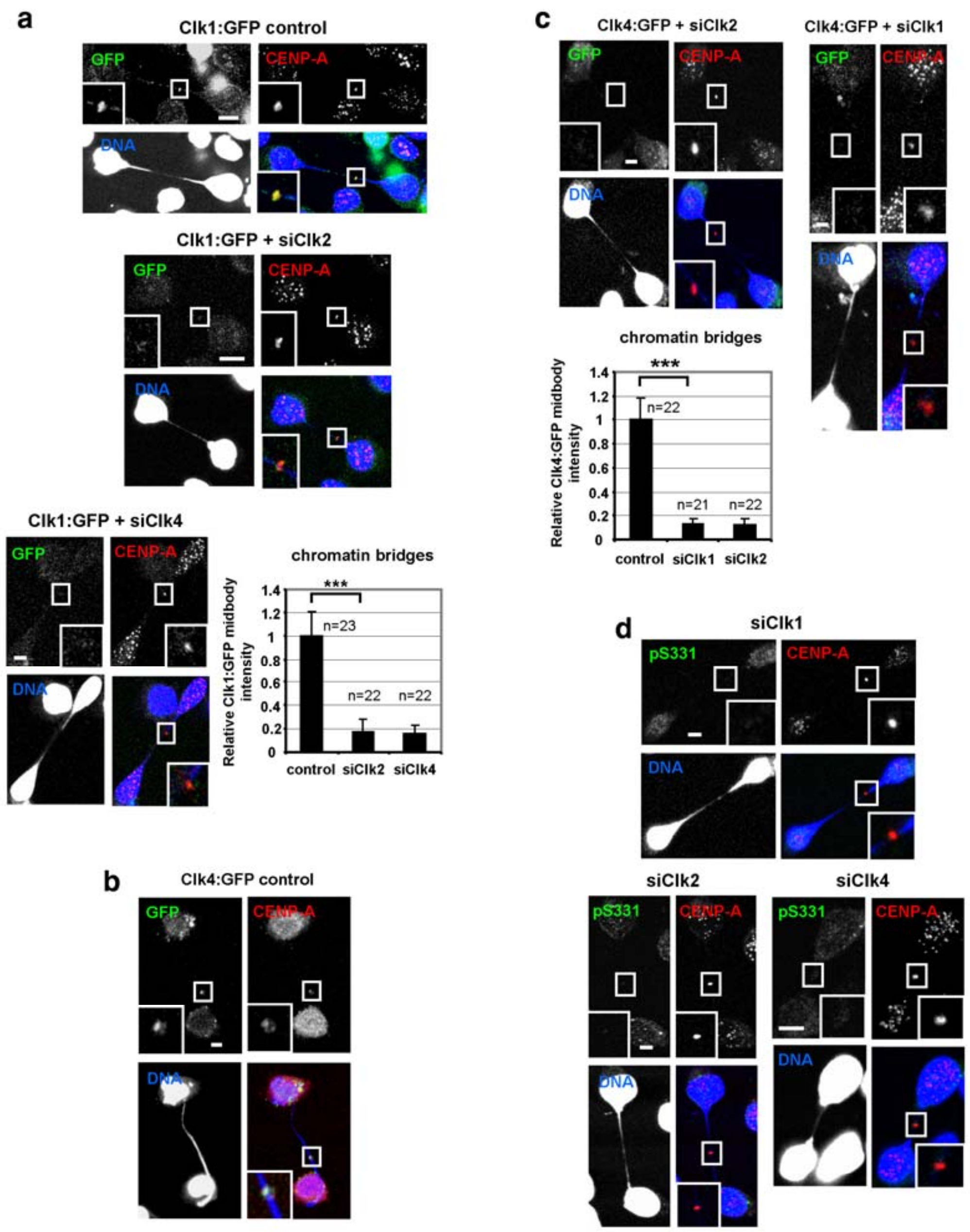

Supplementary Figure 8. Depletion of one Clk protein diminishes localisation of Clk1 or Clk4 at the midbody in cytokinesis with chromatin bridges. (a-c) Cells expressing Clk1:GFP or Clk4:GFP were transfected with negative siRNA (control), Clk1 siRNA (siClk1), Clk2 siRNA (siClk2) or Clk4 siRNA (siClk4). Green, GFP; red, CENP-A; Blue, DNA. Mean midbody intensity of Clk1:GFP or Clk4:GFP is also

shown. Data is from *n* cells from three independent experiments. Values in control were set to 1. Error bars show standard deviation. Three asterisks, $p<0.001$ compared with control. The Mann-Whitney U test was used. **(d)** Aurora B-S331 phosphorylation (pS331) in cells transfected as in **a-c**. Green, pS331; red, CENP-A; Blue, DNA. Insets show magnified midbodies. Bars, 5 μm.

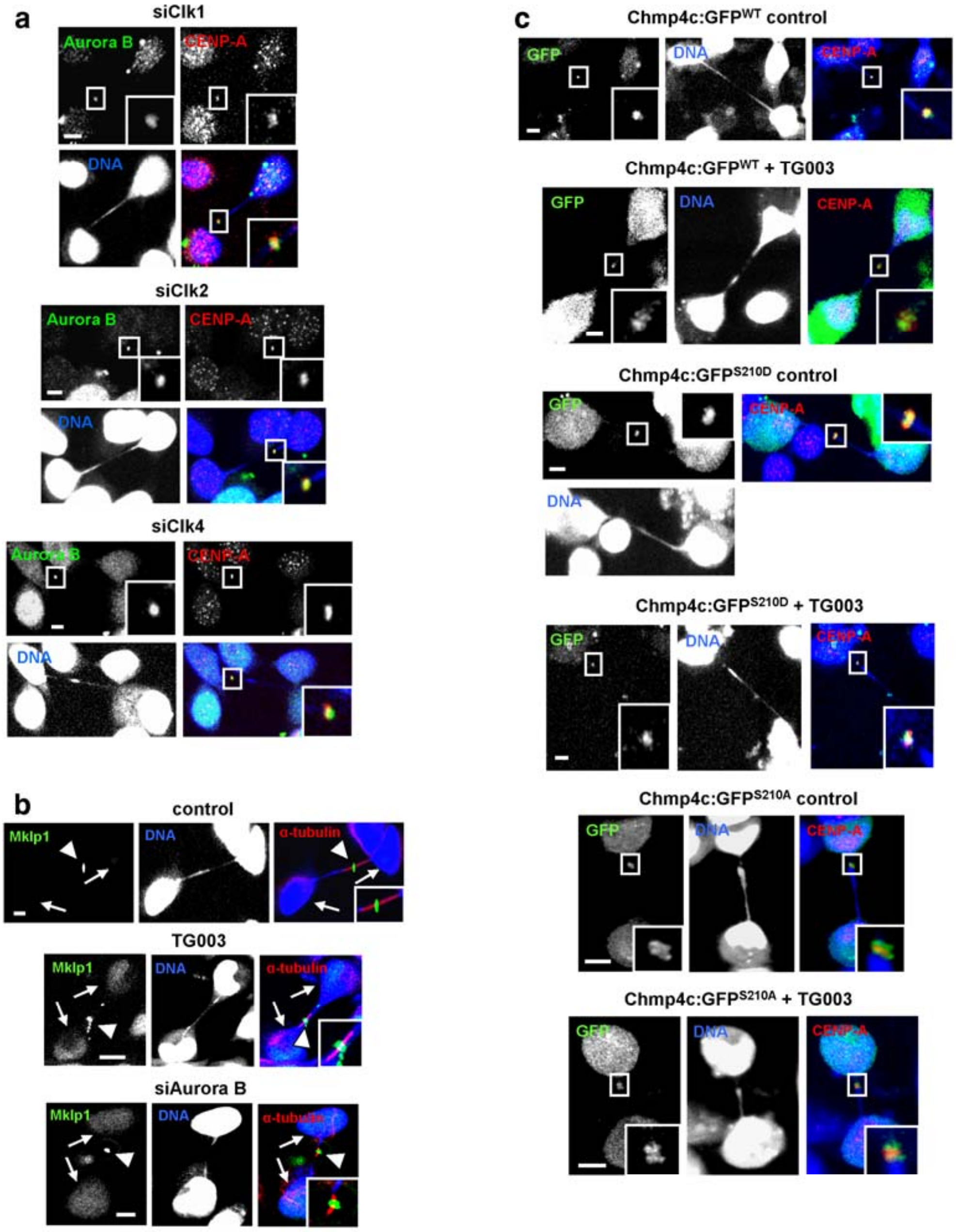

Supplementary Figure 9. The phosphomimetic mutation S210D rescues localization of Chmp4c:GFP to the midbody as a single dot after Clk inhibition. **(a)** Localisation of Aurora B in cells transfected with Clk1 siRNA (siClk1), Clk2 siRNA (siClk2) or Clk4 siRNA (siClk4). Green, Aurora B; red, CENP-A; Blue, DNA. **(b)** Localisation of Mklp1 in the absence (control) or presence of TG003 for 5 h, or

after transfection of cells with Aurora B siRNA (siAurora B). Daughter nuclei are indicated by arrows and midbodies by arrowheads. Green, Mklp1; red, α-tubulin; Blue, DNA. 20 cells from two independent experiments were examined per treatment. **(c)** Cells expressing WT, S210D or S210A Chmp4c:GFP were treated as in **b**. Green, GFP; red, CENP-A; Blue, DNA. 22 cells from three independent experiments were examined per treatment. Insets show magnified midbodies. Bars, 5 μm.
